# Energetics and genetics across the prokaryote-eukaryote divide

**DOI:** 10.1186/1745-6150-6-35

**Published:** 2011-06-30

**Authors:** Nick Lane

**Affiliations:** 1 Department of Genetics, Evolution and Environment, University College London, Gower Street, London WC1E 6BT, UK

## Abstract

**Background:**

All complex life on Earth is eukaryotic. All eukaryotic cells share a common ancestor that arose just once in four billion years of evolution. Prokaryotes show no tendency to evolve greater morphological complexity, despite their metabolic virtuosity. Here I argue that the eukaryotic cell originated in a unique prokaryotic endosymbiosis, a singular event that transformed the selection pressures acting on both host and endosymbiont.

**Results:**

The reductive evolution and specialisation of endosymbionts to mitochondria resulted in an extreme genomic asymmetry, in which the residual mitochondrial genomes enabled the expansion of bioenergetic membranes over several orders of magnitude, overcoming the energetic constraints on prokaryotic genome size, and permitting the host cell genome to expand (in principle) over 200,000-fold. This energetic transformation was permissive, not prescriptive; I suggest that the actual increase in early eukaryotic genome size was driven by a heavy early bombardment of genes and introns from the endosymbiont to the host cell, producing a high mutation rate. Unlike prokaryotes, with lower mutation rates and heavy selection pressure to lose genes, early eukaryotes without genome-size limitations could mask mutations by cell fusion and genome duplication, as in allopolyploidy, giving rise to a proto-sexual cell cycle. The side effect was that a large number of shared eukaryotic basal traits accumulated in the same population, a sexual eukaryotic common ancestor, radically different to any known prokaryote.

**Conclusions:**

The combination of massive bioenergetic expansion, release from genome-size constraints, and high mutation rate favoured a protosexual cell cycle and the accumulation of eukaryotic traits. These factors explain the unique origin of eukaryotes, the absence of true evolutionary intermediates, and the evolution of sex in eukaryotes but not prokaryotes.

**Reviewers:**

This article was reviewed by: Eugene Koonin, William Martin, Ford Doolittle and Mark van der Giezen. For complete reports see the **Reviewers' Comments **section.

## Background

We used to think that if we knew one, we knew two, because one and one are two. We are finding that we must learn a great deal more about 'and'. Sir Arthur Eddington (1882-1944)

### The origin of the eukaryotic cell was a unique event

There is little doubt that all known eukaryotic cells share a common ancestor that arose only once in four billion years of evolution. Common traits range from the conserved position of many introns [1], to the structure of nuclear pore complexes [2], to complex traits such as syngamy and two-step meiosis [3]. It is implausible that all of these shared properties arose by lateral gene transfer (which is inherently asymmetric in mechanism) or convergent evolution (which implies that traits like intron position are dictated by selective constraints, rather than historical contingency). Common ancestry is much the most parsimonious explanation.

However, a single ancestor is perfectly consistent with multiple origins if all 'protoeukaryotic' lines arising later were driven to extinction by fully-fledged eukaryotes already occupying every niche, and if all earlier protoeukaryotes were displaced by modern eukaryotes (or fell extinct for some other reason). This cannot be addressed phylogenetically, as any phylogenetic evidence for their existence is lost. Nor is the fossil record any help. It is hard to distinguish between eukaryotic and prokaryotic microfossils let alone prove the existence of extinct lines of protoeukaryotes. While asserting the unprovable existence of extinct lines of eukaryotes is unsatisfying, if not unscientific, extinction is commonplace, and the argument seems, on the face of it, irrefutable.

But there are several reasons to doubt that prokaryotes have repeatedly given rise to more complex 'protoeukaryotes', which were ultimately all driven to extinction by modern eukaryotes that came to occupy every niche. The periodic mass extinctions of plants and animals, followed by evolutionary radiations of hitherto suppressed groups, are not characteristic of microbial evolution-such radiations explore morphological, not metabolic, space. Moreover, large animals and plants generally have tiny populations in comparison with microbes, and cannot acquire life-saving genes by lateral gene transfer, making animals and plants much more vulnerable to extinction. The continuity of global geochemical cycles over three billion years [4] shows that no major prokaryotic group has been driven to extinction, not even methanogens and acetogens, the most energetically tenuous forms of life. The abundance of apparently parallel niches [5] suggests that extinction is not the rule. Archaea, once believed to be restricted to extreme environments such as hydrothermal vents and salt flats, are common in temperate oceans [6], whereas eukaryotes, long thought to be excluded from extreme environments by their delicate constitutions, are in fact abundant in anoxic conditions [7] and in rivers contaminated with heavy metals [8]. Picoeukaryotes compete directly with prokaryotes in many environments [9], yet neither group has fallen extinct. Extinction seems too facile an explanation to account for fact that all complex life on Earth shares a common ancestor that only arose once. If indeed many other independently arising lineages of protoeukaryotes all fell extinct, more persuasive reasons are needed than simple displacement by more competitive modern eukaryotes.

The existence of a diverse group of morphologically simple eukaryotes that occupy an intermediate niche between prokaryotes and more complex protists refines this point. Described as archezoa by Cavalier-Smith in the 1980s [10,11], the group was seen as primitively amitochondriate protoeukaryotes, living fossils of the prokaryotic-eukaryotic transition [12,13]. Genetic and morphological studies, however, revealed that all known archezoa possessed mitochondria in the past, and lost them via reductive evolution to specialised organelles called hydrogenosomes and mitosomes [14-17]. This is significant in terms of extinction. There are at least 1000 species of simple protist that lack mitochondria, yet all of them evolved by reductive evolution from more complex ancestors, rather than evolving 'up' from more simple prokaryotes. Considered purely in terms of chance, the likelihood of this is around one in 10^300 ^against. Allowing for independent phylogenetic origins on a more realistic 20 separate occasions, the probability is still one in a million. This pattern is unlikely to be chance. Either there was a competitive advantage to reductive evolution (but if so, why should complex aerobic protists displace anaerobic specialists by becoming more like them?) or there was heavy selection against prokaryotes evolving greater morphological complexity. That seems to be true.

### Prokaryotes show no tendency to evolve greater morphological complexity

Despite their metabolic virtuosity, living prokaryotes are barely distinguishable from 3-billion year old microfossils in their morphological appearance [18]. At a molecular level there is no obvious reason for this limitation: bacteria made a start up every avenue of complexity, but then stopped short. There are prokaryotic examples of straight chromosomes [19], DNA recombination [20], multiple replicons [21], introns and exons [22], extreme polyploidy [23], nucleus-like structures [24], internal membranes [25], giant size [26], dynamic cytoskeleton [27], predation [28], parasitism [29], intercellular signalling [30], endocytosis [31], even endosymbionts [32,33]. What prokaryotes lack is the characteristic eukaryotic accumulation of all of these traits at once, typically in much larger cells with complex internal compartments and intracellular transport networks, all encoded by genomes that range freely from bacterial size up to scores of Gigabases, even in protists [34]. The absence of real morphological complexity in bacteria is plausibly ascribed to the dominant mode of prokaryotic evolution: prokaryotes are streamlined by selection for small genomes and fast replication, quickly losing unnecessary genes, and frequently acquiring new genes from the metagenome, when needed, by lateral gene transfer [35,36].

But why are eukaryotes not equally subject to heavy selection for streamlining? Some are, certainly, but many are not, and most eukaryotic streamlining appears to be secondary. Population geneticists ascribe the accumulation of genes in eukaryotes to reduced purifying selection in small populations [37]; but if so, why don't smaller populations of prokaryotes accumulate larger genomes for exactly the same reasons? If the constraint was circular chromosomes [38] why didn't bacteria with straight chromosomes and multiple replicons become complex? If phagocytosis was the critical step [39,40], what stopped wall-less prokaryotes with an incipient capacity for endocytosis (protein uptake) and dynamic cytoskeletons from evolving true complexity? This is ultimately a question about the nature of natural selection. If traits such as the nucleus, phagocytosis and meiotic sex evolved by natural selection acting on ordinary mutations in large or small populations of prokaryotes, and each step offered an advantage, then why did the same traits not evolve repeatedly in prokaryotes, as did the eye [41] in eukaryotes? As noted already, there is no reason to suppose that such protoeukaryotes should have been driven to extinction by more competitive modern eukaryotes; rather, prokaryotes just seem to have no proclivity to explore morphological space.

### Eukaryotes originated in an endosymbiosis between prokaryotes

The fact that all eukaryotic cells either have, or once had, mitochondria, means that the acquisition of mitochondria by some host cell was at the very least an early event in eukaryotic evolution. And large scale, genome-wide phylogenetic studies [42-46] suggest that an endosymbiosis between prokaryotes might well have been the singular event that broke the eternal loop of prokaryotic simplicity.

There is little doubt that the ancestor of the mitochondria was a free-living bacterium, probably most closely related to α-proteobacteria [47] (whatever they were 1.5-2 billion years ago), but its metabolic capabilities are uncertain and disputed [48]. However, given the ubiquitous phylogenetic distribution of anaerobic mitochondria and hydrogenosomes across all the eukaryotic supergroups [49], the most parsimonious answer is that the mitochondrial ancestor was a metabolically versatile, facultatively anaerobic bacterium, perhaps similar to *Rhodobacter *[50].

**Figure 1:**
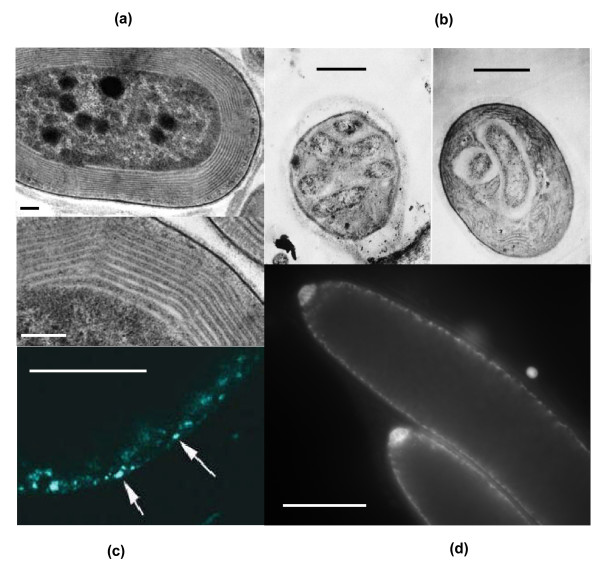
**Genomes and membranes in eukaryotes and prokaryotes**. (a). TEM of cyanobacteria, showing large expansion of bioenergetic membrane surface area as internal thylakoid membranes. However, cyanobacteria are sufficiently small that one or a few copies of the genome (not visible) are sufficient to retain control over chemiosmotic coupling. Scale bars: 50 nm. Reproduced with permission from Miller SR *et al. PNAS *2005, **102:**850-855. (b). TEM of intracellular bacteria living within free-living cyanobacteria (*Pleurocapsa minor*): one of only two known examples of a prokaryote inside a (walled) prokaryote, which must have gained entry without phagocytosis. Scale bars: 1 μM. Reproduced with permission from Wujek D. *Trans Am Micros Soc *1979, **98:**143-145. (c). Multiple copies of nucleoids, each containing the complete genome of *Thiomargarita*, stained with DAPI. Giant vacuole above in black. Scale bar 50 μM. Courtesy of Heide Schultz-Vogt. (d). Extreme polyploidy in *Epulopiscium*, (stained with DAPI) showing peri-membrane location of nucleoids, each genome about 3.8 Mb in size. Scale bar 50 μM. Courtesy of Esther Angert.

The identity of the host cell is even more contentious [51,52]. Most large-scale genomic analyses point to a *bona fide *prokaryote, an archaeon of some sort [42-46], albeit not falling clearly into any modern group, so again its metabolic capabilities are unknown. It is therefore difficult to reconstruct the relationship between the endosymbiont and host cell by phylogenetics alone. A common argument against this scenario (an endosymbiosis between two prokaryotes) is that one prokaryote could not have gained entry to another except through phagocytosis. This argument is refuted by two known examples of prokaryotic cells living within other prokaryotes [32,33] (Figure 1)--plainly it is possible, even if extremely uncommon. Indeed, the very improbability of such an event helps to explain the unique origin of eukaryotes [53]. (It should also be noted that endosymbionts are also known in fungi, despite the fact that fungi are no more phagocytic than bacteria [54].)

Because phylogenetics cannot presently constrain the identity of either host cell or endosymbiont, it cannot give a clear insight into eukaryogenesis--the crossing of that deep gulf between prokaryotes and eukaryotes. One cell inside another cell may have broken the eternal prokaryotic loop, but this situation is far removed from the morphological complexity of even the simplest eukaryotic cell. Is it possible to gain an insight into what happened next without the aid of phylogenetic reconstruction?

## Cell fusions best explain the accumulation of eukaryotic traits

**Figure 2:**
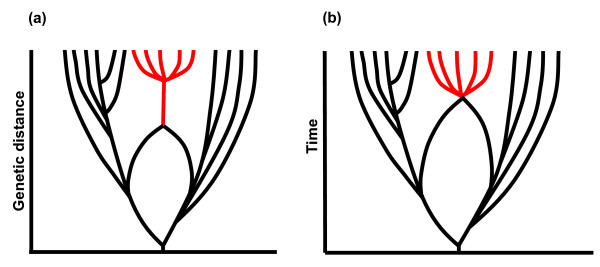
**Endosymbiotic origin of eukaryotes by time and genetic distance**. Schematic depiction of the chimeric origin of eukaryotes (in red) by (a) genetic distance and (b) time. Bacteria and archaea are shown to the left and right, respectively. Reticular networks of lateral gene transfer are not shown for simplicity, but characterise prokaryotic evolution. In (a) the unbranching red trunk depicts the prokaryote-eukaryote transition without any successful speciation (as attested by the absence of true evolutionary intermediates; see text) across the long genetic distance from an endosymbiotic origin in prokaryotes to LECA. In (b) the absence of this unbranching trunk depicts the short timescale and rapid evolution of LECA, driven by endosymbiotic gene transfer, a high mutation rate, cell fusions and genome doublings, accumulating traits within a single small population.

Any hypothesis for the origin and evolution of eukaryotes must explain why prokaryotes show little tendency to evolve morphological complexity; why the Last Eukaryotic Common Ancestor, LECA, was morphologically complex; and why no true evolutionary intermediates exist, despite the niche being viable, and indeed filled with thousands of simple protists [10,11]. Prokaryotes and eukaryotes both speciate profligately, meaning that genetic variation falls into innumerable discrete clusters, which correspond to species as defined by Darwin and elaborated by Mallet [55]. Despite this universal tendency to vary and diverge, there was no successful speciation across the prokaryote-eukaryote transition. That is to say, there are no surviving evolutionary intermediates--no 'early branching' species, equivalent to the discredited archezoa, despite the great evolutionary distance crossed. If the arguments marshalled here are correct, the trigger, or starting point, for eukaryogenesis was an endosymbiosis between prokaryotes: a prokaryote within a prokaryote, lacking a nucleus or any of the other signature eukaryotic traits. In contrast, LECA was recognizably eukaryotic, with a nucleus, straight chromosomes, introns and exons, a cell cycle, meiosis and mitosis, dynamic cytoskeleton, motor proteins and intracellular transport mechanisms, endomembrane systems and mitochondria. All of these traits apparently evolved in a population of cells that never diverged to form a successful early-branching species. There are no evolutionary intermediates with a nucleus but no endoplasmic reticulum, or mitochondria but no nucleus, or a dynamic cytoskeleton but no meiosis. Over this long evolutionary distance, the prokaryote-eukaryote transition, the tree of life is neither a branching tree nor a reticular network, but what amounts to an unbranching trunk (Figure 2).

Only certain forms of inheritance could begin to explain such a trunk. Being inherently asymmetric, lateral gene transfer surely cannot explain the universality of eukaryotic basal traits: it is far more likely to give rise to the pattern that is actually seen in prokaryotes, in which different species possess different traits, and none possesses them all. But reciprocal sex (or some form of cell fusion) can readily explain the accumulation of eukaryotic traits. If so, then sex must have arisen very early in eukaryotic evolution, as is borne out by phylogenetic analyses [56-58]; but here this is a logical inference, not an observation.

Likewise, the evolution of eukaryotes must have been rapid, in a small population. If the population had been large, the individual cells should have been successful (stable and viable) and should have become structured in space. Spatial structuring of stable populations should have led to divergence and speciation, at least some of which ought to have permanently occupied the 'archezoan niche' (that filled by morphologically simple, 'primitive' eukaryotes). They did not, despite the viability of the niche, so the population must have been small. For similar reasons, evolution had to be fast. If the pace of evolution was slow, the individual cells should have been successful (stable and viable) and so should have become structured in time and space: they should have undergone speciation. The fact that they did not again implies instability. Finally, the requirement for sex also implies that the population was small; large stable populations should speciate, as indeed happened immediately after the crystallization of LECA, with a near-immediate radiation of the eukaryotic supergoups [2]. These surmises are consistent with calculations based on intron density, which also suggest a tight bottleneck at the origin of eukaryotes [59].

It is thus likely that eukaryotes evolved from a small population of prokaryotes with endosymbionts: unstable, rapidly evolving, protosexual cells. These circumstances enabled the ubiquitous acquisition of traits, but for some reason, unlike bacteria, there was little selection pressure to lose them again.

### Mitochondria solve the riddle of eukaryotic origins

I shall argue in this paper that a singular event--the acquisition of mitochondria--transformed the selection pressures acting on the prokaryotic host cell. Mitochondria and specifically mitochondrial genes, enabled the surface area of bioenergetic membranes to be increased over several orders of magnitude, which in turn permitted expansion of the host cell genome capacity over several orders of magnitude [34]. That lifted the ceiling on prokaryotic genome size, eliminating the selection pressure to lose genes, but did not drive the accumulation of genes; it was permissive, not prescriptive. Two factors did drive the expansion of eukaryotic genomes, and I shall argue that both were linked to the bombardment of DNA from the mitochondria to the host cell: first, the host cell acquired bacterial genes by standard nonhomologous recombination without any requirement to lose them again; and second, the bombardment of mitochondrial DNA produced a high mutation rate, which was offset by cell fusion and masking with new genes--the origin of sex.

## Results and Discussion

### Energy per gene

Prokaryotes and eukaryotes respire at roughly a similar rate--the mean metabolic rate is 0.19 ± 0.5 W g^-1 ^(1 Watt = 1 J sec^-1^) in prokaryotes (based on a mean of 55 samples) versus 0.06 ± 0.1 W g^-1 ^in protozoa (based on 12 samples) [60,61]. However, these mean metabolic rates per gram conceal a host of subtleties, as eukaryotic cells are, on average, much larger than prokaryotes, with a mean mass of 40,100 × 10^-12 ^g for eukaryotes versus 2.6 × 10^-12 ^g for prokaryotes. The mean metabolic rate per cell is therefore 0.49 pW for bacteria, versus 2,286 pW for protozoa. In other words, metabolic rate per gram is not particularly different (a factor of 3) but cell volume is very different (a factor of 15,000), so an average protozoan has nearly 5000 times more metabolic power (W) than a single bacterium.

This additional metabolic power supports additional genes and DNA in eukaryotes, as can be seen from a consideration of energy per gene. The metabolic power per Mb of DNA is similar in bacteria and protozoa, to within an order of magnitude. Assuming 6 Mb of DNA, an 'average' bacterium has about 0.08 pW Mb^-1^. The largest bacterial genomes, around 10 Mb, would have about 0.05 pW Mb^-1^, while the smallest free-living bacteria, with around 1.5 Mb, would have about 0.3 pW Mb^-1^, in each case assuming a similar cell size (but see below on scaling). These values are in the same range as those calculated for specific bacteria and discussed elsewhere [34].

Protists exhibit an extraordinary range of genome sizes, from bacterial sizes up to around 100,000 Mb [62,63]. While many fungi and small protists have genome sizes in the range 10-40 Mb [64,65], fungal genome sizes range up to 1000 Mb [65]. The mean for mitochondriate phagotrophs is about 700 Mb [63], ranging up to 10,000 Mb [Jékely and Cavalier-Smith, personal communication]. Mean genome sizes for algae are larger again, at around 3000 Mb for *Cryptophycaea *and *Dinoflagellates*, ranging up to 10,000 and 100,000 Mb, respectively [66,67]. Taking an 'average' of 3000 Mb, protists would have a power of 0.76 pW Mb^-1^. Thus, despite the fact that bacteria have a faster metabolic rate *per gram *than protozoa, their small size disguises the fact that the power dedicated to each Mb of DNA has remained roughly constant, to within an order of magnitude. If the nuclear genome were smaller, say 100 Mb, then the energy per Mb of DNA would expand to 22 pW Mb^-1^. A genome of 10 Mb would give the protist a power of 228 pW Mb^-1^, nearly 3000 times greater than the bacterium. Having said that, the actual metabolic rate, and the number of mitochondria required to support it, is very much lower than the protozoan average in such small protists. *Ochromonas*, for example [34], with a haploid genome of 300 Mb, has a power of only 0.04 pW Mb^-1^, squarely in the bacterial range. The point is that eukaryotes and prokaryotes often have a similar metabolic power per Mb of DNA, but prokaryotic genome sizes are limited to 10 Mb or less, whereas eukaryotic genome sizes can expand freely to 100,000 Mb, and in doing so are plainly not constrained by energetics.

This conclusion remains true even when considering the genomic weight of mitochondrial DNA (mtDNA). Over evolutionary time, mtDNA has been whittled away to between 6 Kb and 77 Kb in protozoa [68]. Taking an average mitochondrial genome of 30 Kb in 200,000 copies (as in large amoebae [69]), the total mtDNA content per cell is 6000 Mb, twice the size of the average haploid nuclear genome, or 9000 Mb in total for the 'average' protist. The genomic power now corresponds to 0.25 pW Mb^-1^; still more than most bacteria, yet unlike bacteria sustaining a nuclear genome of 3000 Mb.

**Figure 3:**
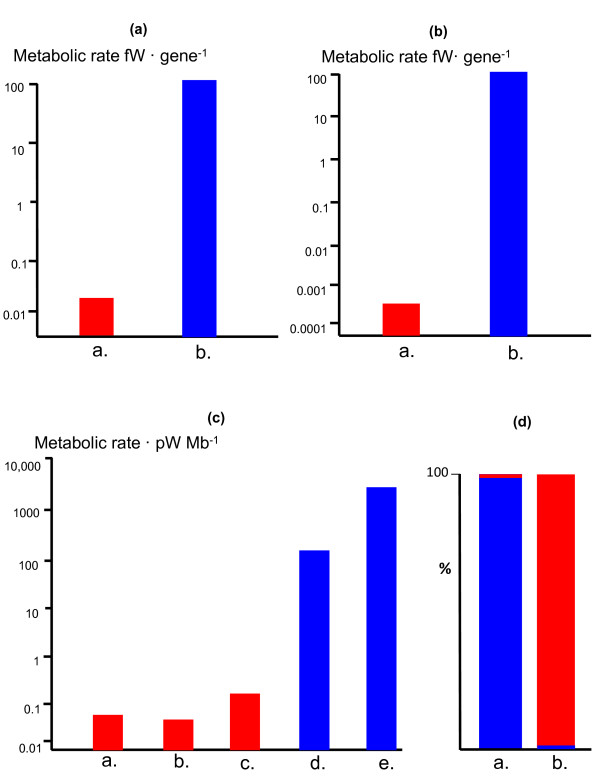
**Energetics of genome size in eukaryotes and prokaryotes**. (a). Mean energy per gene in prokaryotes versus eukaryotes equalised for genome size. Prokaryotes in red, eukaryotes in blue. Note log scale. (b). Mean energy per gene in prokaryotes versus eukaryotes equalised for genome size and cell volume; see text. Prokaryotes in red, eukaryotes in blue. Note log scale. (c). Power per haploid genome (energy per gene x number of genes in one haploid genome) in **a**. *E. coli *(metabolic rate taken from Ref 60); **b**. *Thiomargarita *(metabolic rate taken from Ref 83); **c**. *Epulopiscium *(metabolic rate taken, conservatively, to be equal to *A. proteus*); **d**. *Chlamydomonas *(metabolic rate taken from Ref 60); **e**. *Amoeba proteus *(metabolic rate taken from Ref 61). Note log scale and broad agreement with derived mean values in (a) and (b). (d). Proportion of genome free to vary (in red) equalised to 100,000 Mb in **a**. *Epulopiscium *and **b**. *Amoeba proteus*. Blue bar depicts proportion of total DNA content required for maintaining control over cytoplasm using an equal copy number (26,000; scaled from values given in text) of **a**. a 3.8 Mb genome; and **b**. a 30 Kb mitochondrial genome. 'Free to evolve' means genomic capacity beyond a standard prokaryotic genome required to govern a fixed volume of cytoplasm.

The situation is more pronounced in terms of gene number. An average bacterial genome (such as *E. coli*) contains nearly 5,000 genes, compared with some 20,000 in an average protist, such as *Euglena *ranging up to 40,000 in *Paramecium *[62,63,70,71]. At a metabolic rate of 0.49 pW per cell, a bacterium with 5000 genes has only 0.1 fW per gene. Smaller bacteria, with around 2,500 genes, have a power of 0.2 fW per gene. Larger bacteria, with around 10,000 genes are surely close to a lower functional limit, with a power of just 0.05 fW per gene. In contrast, at a power of 2,286 pW per cell and 20,000 genes, an average protist has about 115 fW per gene, over 1000-fold more energy per gene than an average bacterium, and more than 2000-fold more than a large bacterium. Increasing bacterial gene number further, without an equivalent increase in ATP synthesis, is unlikely to be sustainable; and increasing bacterial gene number up to the eukaryotic mean of 20,000 genes would give bacteria nearly 5000-fold less energy per gene (Figure 3). This perspective helps explain why both gene number and genome size remain within tight limits across prokaryotes. The lower limit is set by the number of genes needed for a free-living existence; the upper limit, arguably, by energetic constraints. I will develop this argument further below.

It is notable that eukaryotes support, on average, around 500 times more DNA than prokaryotes but only four times as many genes. Non-coding DNA is relatively cheap, but its maintenance still has an energetic cost, as well as slowing replication [72,73]. In general, DNA replication consumes around 2% of bacterial energy budget. Increasing DNA content 10-fold would still only consume 20% of the cell's energy budget, no doubt affordable. But increasing DNA content to 3000 Mb, the protist mean, would require the bacterial energy budget to be raised 12-fold, a serious cost in its own right. And if the genome expanded to 100,000 Mb, the upper reaches of protistan genomes, the energy budget would need to be raised 400-fold. It is therefore hardly surprising that bacteria maintain a high gene density, 500-1000 genes per Mb (compared to the eukaryotic average of around 12 genes per Mb). They do so by eliminating most intergenic and intragenic material that might happen to arise [72-74] (preventing the potential evolution of regulatory elements and microRNAs), by organising genes into operons, and by restricting the median length of proteins [75]. In contrast, eukaryotes have invested freely in regulatory microRNAs [76].

But the energetic constraints on DNA content are relatively forgiving compared with the far heavier energetic constraints on protein synthesis, and therefore on the energy per gene. Given that protein synthesis accounts for a remarkable 75% of the total energy budget of growing microbes [77], there is a near linear relationship between the number of genes and the energetic cost, a cost that is made tangible by ribosome numbers. *E. coli*, for example, has up to 13,000 ribosomes, compared with 13 million on the rough ER alone in a liver cell--1000-10,000 times more [34]. This value corresponds closely to the abundance of energy that eukaryotes are able to dedicate to the expression of their additional genes--nearly 5000-fold more when normalised for genome size.

This additional 1000-5000-fold more energy per gene helps to explain why the dynamic eukaryotic lifestyle, including archetypal traits like phagocytosis, never arose in prokaryotes. Phagocytosis is not unknown in amitochondriate eukaryotes such as 'archezoa' that lost their mitochondria secondarily. However, the energetic costs for the *de novo *'invention' of complex traits like phagocytosis must far exceed the costs of simply inheriting a functional system. Thus it might well require mitochondria to 'invent' phagocytosis, but once invented it is possible to evolve reductively in certain environments, while retaining phagocytosis. For example, *Entamoeba histolytica *is an amitochondriate phagotroph with nearly 10,000 genes [78], equivalent to a large bacterial genome. Characteristically for parasites it has undergone reductive evolution from more complex mitochondriate ancestors, and today lacks pathways for amino acid biosynthesis, purine and pyrimidine synthesis, fatty acid biosynthesis and TCA cycle [78]. In other words, by cutting back on the costs of intermediary metabolism, *E. histolytica *has been able to maintain its expensive phagocytic machinery (albeit this is pared down too relative to large free-living amoebae). Thus the acquisition of mitochondria enabled an accumulation of DNA and genes, allowing profligate experimentation with new protein folds, new proteins, gene families and regulatory elements, without the heavy bacterial selection pressure to lose them all again. I am proposing that this freedom permitted the *de novo *evolution of complex traits like phagocytosis, not possible without mitochondria. But once in existence, there was nothing to stop complex eukaryotes evolving reductively in certain environments, while retaining some complex traits like phagocytosis. In these cases, their energy per gene (and genome size) is no longer greater than bacteria; but their ability to phagocytose enables them to compete successfully in 'bacterial' niches, despite being metabolically less versatile.

Thus prokaryotes exist at the bottom of a deep canyon in the energy landscape, from which they have never escaped, except at the origin of the eukaryotic cell [34]. Eukaryotes have 1000-5000-fold more energy to burn per gene at least in part *because *they are larger. Obviously, larger cells need to generate more energy per gene to sustain themselves. Bacteria, in contrast, are usually small. I shall argue that the reason bacteria are small is that large bacteria cannot generate a lot more energy per gene. On the contrary, they generate a lot less. The problem relates to scaling.

### The issue of scaling

The problem of scaling does not relate simply to surface-area to volume constraints. If the linear dimensions of the cell are increased 25-fold--corresponding to a 15,000-fold increase in cell volume, equivalent to the mean difference in cell volume between prokaryotes and eukaryotes in this study--the surface area of the plasma membrane increases 625-fold. The deficit of prokaryotes relative to eukaryotes is then apparently reduced to a mere 8-fold--surely not an insurmountable difference, especially if bacteria internalise respiration to some extent on invaginated membranes, as happens in more complex prokaryotes like cyanobacteria, with their internal thylakoid membranes (Figure 1).

Of course, if the giant bacterium is metabolically active and composed of proteins, then demand for protein synthesis would rise by 15,000-fold (the increase in cell volume) and this increased demand must be met by a 625-fold increase in ATP production. This, however, is equally true for eukaryotes, which still must synthesise the proteins to fill their volume. Thus a giant bacterium respiring over its plasma membrane would not be penalised much relative to a large eukaryote respiring internally. It is true that there are few limits to the number of mitochondria that a eukaryote could accumulate in principle; but the same might seem to apply to internal membranes in bacteria. However, this is not the case.

The catch here lies in the assumption that ATP synthesis per unit membrane is a constant, without a cost in protein synthesis or distribution. Obviously this is not true-scaling is not transubstantiation. The very act of increasing cell volume and surface area on such a scale in turn demands some sort of compensation in terms of ribosome numbers, genome copies and protein synthesis, otherwise the scaled up bacterium is merely an empty bag, not a living cell. ATP synthesis depends on respiratory proteins implanted into the membrane at high concentration. If the rate of protein synthesis is unchanged, but the surface area of plasma membrane is increased 625-fold, then ATP synthesis per unit area must fall by 625-fold (not even allowing for inefficiencies in intracellular transport). It is impossible for a bacterium to step up the rates of transcription and translation by 625-fold from a single genome; the only reasonable solution to scaling up on such a scale would be to scale up the total number of genomes accordingly--most reasonably, assuming that streamlined bacteria already approach the limits of efficiency, by a factor of 625-fold. If so, then the energy available per gene remains unchanged, and the cell would have 625 copies of its genome, each controlling an equivalent volume of cytoplasm and area of bioenergetic plasma membrane.

But what of the internal volume? There are two extreme possibilities: either the internal volume is metabolically inert, in which case the cell is not equivalent to a eukaryote; or it is metabolically active. In the first case, the internal volume is like a balloon, with a thin skin of active cytoplasm surrounding an inert space, metabolically equivalent to a giant vacuole. It has the same energy per gene as any other bacterium, but is faced with a serious challenge when it comes to cell division, as bacterial division usually depends on a Z-ring with a maximal diameter of about 1 μM [79]. Giant bacteria are obliged to produce endospores or to divide reductively (giving rise to clumps of cells that have been mistaken for animal embryos [80]). Either way, normal binary fission will not do; scaled-up bacteria are obliged to evolve novel forms of division.

The second possibility is for the inner volume to be metabolically active, as is the case in eukaryotes. But the scaled up bacterium would now face an even more serious problem: rates of transcription and translation could not be scaled-up 15,000-fold to service the increased cell volume, except by scaling up genome number, as before. But scaling up genome number 15,000-fold with only a 625-fold increase in ATP availability would reduce the energy per gene a further 25-fold. Equalising for both gene number (4,600-fold less energy per gene in bacteria) and cell volume gives a factor of 115,000 times less energy per gene than the comparable eukaryote. If the bacterium were scaled up to the size of a large amoeba (a 50-fold increase in linear dimension) then the energy per gene would be a remarkable 230,000-fold less than the amoeba (Figure 3). This is the real scale of the energy canyon that has forced prokaryotes to remain prokaryotic for 4 billion years.

This line of argument is substantiated by several examples of extreme polyploidy in giant bacteria (Figure 1). The best example is *Epulopiscium*, a giant Gram-positive bacterium that lives only in the anaerobic guts of the surgeonfish. Growing up to 0.6 mm in length, this cigar-shaped bacterium has as many as 200,000 copies of its full genome [23], all of them associated with the plasma membrane. The inner volume is metabolically relatively inert, and is ultimately filled with daughter cells growing within--each with their own plasma membranes and associated genomes [81]. A second example is *Thiomargarita*, an even larger, free-living γ-proteobacterium that traps nitrates from up-welling currents in giant internal vacuoles [82]. Here the cytoplasm is a thin active layer, again exhibiting extreme polyploidy--in this case around 8,000-17,000 copies of the full genome [Heide Schultz-Vogt, personal communication; Figure 1]. In both these examples the number of genomes is very much in line with theory; and in both cases the internal volume is metabolically quite inert, totally so in the case of *Thiomargarita *[82,83].

Taking into consideration the energetic costs of expressing these multiple genomes (each one 3-4 Mb in size) the energy available per gene falls well within the prokaryotic range--exactly as would be predicted given the metabolic inertia of the inner volume--several orders of magnitude below eukaryotic values (Figure 3). In the case of *Epulopiscium*, with 200,000 copies of its 3.8 Mb genome, a single bacterium must sustain an extraordinary 760 Mb of DNA just to get through its life cycle. Each genome governs a 'bacterial' volume of cytoplasm in a bacterial fashion (without eukaryotic active transport mechanisms) and there is no spare bioenergetic capacity to evolve more genes, or express them with eukaryotic profligacy. Despite their giant size and their prodigious quantity of DNA, giant prokaryotes remain, in all other ways, prokaryotic. Given that cell division is much more complicated than in smaller bacteria, it is not surprising that giant bacteria only flourish in marginal ecosystems.

But why are eukaryotes not weighed down by the colossal genomic weight of extreme polyploidy? The answer is that they are not immune to the problem--it is simply that the nature of the ploidy has changed as a direct consequence of endosymbiosis. Rather than extreme polyploidy, eukayotes exhibit extreme genomic asymmetry.

### Only endosymbiosis can fashion giant nuclear genomes

Endosymbiosis characteristically results in the reductive evolution of endosymbiont genomes [84]. In the case of the bacteria that eventually became mitochondria, almost the entire genome was either lost or transferred to the nucleus, leaving only a tiny residual genome in most cases (and nothing at all in almost all hydrogenosomes [85]). But mitochondria are by no means unique in this regard. Chloroplasts, too, have lost almost all the genes required by free-living cyanobacteria [86]. Other bacterial endosymbionts living inside eukaryotic cells, such as *Buchnera *[87,88]*Wolbachia *[89], *Rickettsia *[90] and *Carsonella *[91] have retained only stumps of genomes; in the case of *Carsonella*, smaller than many plant mitochondrial genomes [91]. This process probably reflects competition between individual endosymbiotic cells for succession to the next generation. The fastest replicators, typically with the smallest genomes and lowest demand for *de novo *protein synthesis, prevail. The outcome is that unnecessary genes are jettisoned and the genome is gradually pared away. But this process--no more than standard practice for populations of endosymbionts--has the most profound repercussions for the host cell.

The genetic machinery of mitochondria is often thought of as a highly redundant system-hundreds or thousands of copies of mtDNA in every cell, encoding just a handful of respiratory proteins, plus the tRNAs and rRNAs needed to express these proteins *in situ*. Surely, the argument goes, it would be economically more rational to move all these mitochondrial genes to the nucleus, manufacture all mitochondrial proteins on cytosolic ribosomes, and import the proteins into the mitochondria, as more than a thousand are in any case; and as happens without exception in all other membrane systems, such as the endoplasmic reticulum. Of course, this argument only makes sense if there are no countering benefits to the mitochondrial genes remaining *in situ*; and there almost certainly are some important benefits (see below). But in any case, the current arrangement is only uneconomic in relation to a perceived ideal. The relevant comparison is not to a situation in which all mitochondrial genes reside in the nucleus, but to a situation in which all mitochondrial genes reside in the mitochondria--which is analogous to the state of extreme polyploidy in giant bacteria.

In comparison to *Epulopiscium*, with its 760,000 Mb of DNA, a large eukaryote with 200,000 copies of an average protist mitochondrial genome (30 Kb) needs to support only 6000 Mb of DNA. All the rest of the mitochondrial DNA that is lost altogether or transferred to the nucleus as pseudogenes is by definition no longer needed *in situ*, and so is free to vary--to evolve, to encode new proteins with new properties. Equalising for a total genome size of 100,000 Mb, more than 99% of the DNA (that in the nucleus) is essentially free to evolve in the eukaryote, compared with less than 1% in the giant bacterium, with its requirement for many copies of its full genome (Figure 3).

There are two critical points here. The first is that the total amount of DNA sustained by the single cell, and the total amount of protein expressed, could easily be the same in both cases; what has changed is the distribution of DNA and variety of protein within the cell. Eukaryotes exhibit extreme genomic asymmetry, with lots of tiny mitochondrial genomes sustaining a massive nuclear genome. This arrangement depends on cytoplasmic inheritance, with independent replication machinery residing within each endosymbiont, at least during the early phase of eukaryotic evolution. Cytoplasmic inheritance is not possible in the case of extreme polyploidy, where the individual genomes are not independent cellular entities and so cannot partition themselves autonomously within the cell or compete among themselves for succession. During cell division a small proportion of genomes are distributed to daughter cells (1-2% in the case of *Epulopiscium*), and then clonally amplified during daughter cell growth, a process repeated generation after generation. Such clonal amplification makes extreme polyploidy inflexible--essentially unevolvable--and all the copies of an identical genome must remain exactly that--copies of an identical genome, with no potential to lose genes. The acquisition of plasmids might seem to be a way out, but it is not (see below).

Second, in eukaryotes, the process of endosymbiotic gene loss is slow and evolvable (and practically inevitable), with the potential to slowly accumulate large changes over many generations. Because there are so many endosymbionts in eukaryotic cells, even trivial changes in mitochondrial genome size can be significant and selectable at the level of the whole cell. The point is that, as functions are lost (they need to be lost--if they are simply transferred to the nucleus, there is no net gain) there is a net fall in *required *protein synthesis, saving the host cell a great deal in ATP synthesis. But because the endosymbionts ultimately produce ATP for the host cell, and are just as effective at this despite the loss of genes (as they specialise as energy-transducing organelles) there is in effect a net gain in ATP availability, which can be spent on other projects about the cell, such as a dynamic cytoskeleton, at no net cost. There are two very significant points about mitochondria here that do not apply to all endosymbionts--first, they produce ATP for the host cell; and second, the process of endosymbiotic gene loss ultimately cost their independence as cells, and enabled their specialisation as organelles. Specialisation (via selection) for ATP synthesis is crucial, as it means that most other endosymbiont functions can be lost altogether, and the energetic savings diverted into host cell proteome projects.

Consider what might happen if 5% of each endosymbiont genome was initially dispensable, because the symbiont relies on the host cell for some metabolites. In a host cell equivalent *Epulopiscium*, the total amount of DNA sustained by the cell would fall from 760,000 to 720,000 Mb. In terms of genes, if the endosymbionts no longer needed to express 200 out of 4000 proteins, the total protein expression in the cell as a whole would fall by 40 million proteins. In terms of ATP requirements, the ATP cost for transcription and translation of a single peptide bond is a minimum of five ATPs [92], or 1,250 ATPs for a single polypeptide of median bacterial length of 250 amino acids [93]. If each polypeptide were present in a conservative 1000 copies, the total ATP savings would be 50 trillion (50 × 10^12^) ATPs over a 24 hr lifecycle (equivalent to *Epulopiscium*), or 20 million glucose moieties per second.

If such energy savings were redirected to the fabrication of a dynamic ATP-consuming cytoskeleton in the host cell, they could (in principle, if not in practice) fuel the *de novo *synthesis and self-assembly of 800 microns of actin filaments *every second*. Such dynamism is a small evolutionary step in terms of genetic mutation, but is energetically prohibited in giant bacteria that are unable to dispense with other genes via the reductive evolution of cytoplasmic inheritance. But reductive evolution, by permitting the evolution of energetically expensive intracellular transport systems, enables the loss of endosymbiont genes, affording further energy savings. Thus the whole process feeds on itself, paring away at symbiont genomes, and enabling the accumulation of more DNA, new genes and gene families in the host cell genome, with no net energetic cost. At each step, the host cell and endosymbiont both benefit, the endosymbiont steadily losing its autonomy as it becomes integrated into the host, in the end becoming an organelle-mitochondria.

### Genome outposts are required for major expansion of oxidative phosphorylation

Importantly, the end of this process of gene transfer is not the complete loss of mitochondrial genes, but the paring away of the mitochondrial genome to a functional minimum, invariably encoding the same core group of integral membrane proteins, subunits of the respiratory chain [47,68]. Exactly why this same core group is always retained is still disputed, but the Co-location for Redox Regulation (CoRR) hypothesis is by far the most persuasive explanation, being both necessary and sufficient to account for the retention of mitochondrial genes, along with the ribosomes needed to express them on site [94-96]. In essence, genome outposts and ribosomes are needed in the immediate vicinity of the bioenergetic membranes to enable swift and local responses to changes in membrane potential and electron flux in the face of abrupt changes in substrate availability, oxygen concentration (or redox state more generally) and ATP demand. The extreme mitochondrial membrane potential sets the mitochondrial inner membrane apart from any other intracellular membrane system, such as the endoplasmic reticulum [34]. A membrane potential of 150 mV across a 5 nm membrane gives a field strength of 30 million Volts per metre, equal to that discharged by a bolt of lightning. Mistakes can be penalised in a matter of seconds to minutes by free-radical leak, loss of cytochrome c and falling ATP levels--the archetypal trigger for controlled cell death across eukaryotes, from single-celled algae to plants and animals [97-99]. Thus any failure to control mitochondrial membrane potential is punishable by sudden death, which is avoidable because mitochondrial genes enable swift and local compensation at the level of respiratory protein expression. Competing hypotheses for the retention of mitochondrial genes (such as the hydrophobicity hypothesis [100]) are far from mutually exclusive: if importing highly hydrophobic proteins into the mitochondria is laborious and slow, yet there is a requirement for rapid and local responses in gene expression to changes in membrane potential, then hydrophobicity becomes merely a subclause of the CoRR hypothesis.

Mitochondrial genes are undoubtedly necessary for oxidative phosphorylation in eukaryotic cells. A large body of data shows that the rate of respiration depends on the copy number of mitochondrial DNA (mtDNA), with active cells having more copies of the genome [101-104]. Cells depleted in mtDNA have a low respiratory capacity [105], while mutations that cause mtDNA depletion are typically associated with mitochondrial diseases [106]. The rate of transcription of mitochondrial genes, notably ND5, controls the overall rate of assembly of respiratory complexes [107,108]. Presumably, the reverse is true for prokaryotes: in the absence of local genome outposts, giant bacteria could not respire across a significantly wider area of bioenergetic membrane, whether internal or external. All known examples of giant bacteria do indeed have multiple genomes, invariably placed close to the plasma membrane (rather than distributed randomly across the cell) suggesting that local genome outposts are necessary for respiration.

The fact that the plasma membrane is a continuous unbroken sheet in giant bacteria (rather than discrete mitochondria) does not detract from this argument. Proton diffusion and conductivity is extremely high, such that the membrane potential would equalize across the entire surface area in essentially zero time [109]. In contrast, electron flux depends on the concentration of substrates such as NADH, ADP, Pi and oxygen, all of which diffuse orders of magnitude more slowly. Electron flux also depends on the expression and activity of respiratory proteins, which is slower still. Equalisation of membrane potential could therefore drive reverse electron flow, high ROS leak and futile cycling in different regions of the membrane, unless gene expression was sensitive to local changes in redox state, and so compensated for the differences. That requires local genomes encoding the requisite proteins, transcription sensitive to local redox state, and translation coupled directly to membrane insertion; hence the need for multiple local genome outposts.

**Figure 4:**
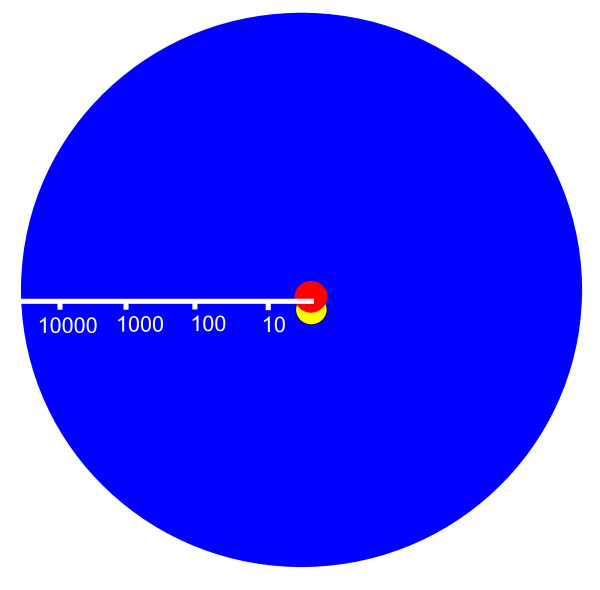
**Volume of cytoplasm controlled by a single genome**. The long reach of the eukaryotic gene. Mean eukaryotic cell volume is 15,000 times greater than mean bacterial cell volume (red circle), and is controlled by a single nuclear genome. In the case of *Thiomargarita *(yellow circle) the cell volume is larger than most eukaryotic cells but is mostly filled with inert vacuole. The band of active cytoplasm contains multiple nucleoids, each one governing a volume of cytoplasm equivalent to a single *E. coli *cell, hence volume per gene is prokaryotic.

Given the size similarity of mtDNA to bacterial plasmids, it might seem possible to control respiration over a wide area of bioenergetic membranes by co-locating plasmids encoding all the genes needed for oxidative phosphorylation. There are various problems with plasmids, which have been discussed in detail elsewhere [34]. However, from the perspective developed here, plasmids could not enable the scaling up of bacteria to eukaryotic size. This is because plasmids cannot meet the general transcriptional and translational requirements of giant bacteria, whereby a single genome controls a roughly fixed volume of cytoplasm in the absence of dynamic transport networks [110]. If there were a single central genome (a standard prokaryotic genome) with hundreds or thousands of large plasmids controlling respiration across a large surface area of membranes, the cell would have plenty of ATP, but would still be unable to distribute any other proteins or substrates about the cell in the absence of highly evolved transport networks (which would take generations to evolve). The cell would fail and die. This problem does not arise if full genomes are distributed throughout the cell, as each genome provides both the energy and proteins required to control a 'prokaryotic' volume of cytoplasm (Figure 4). In other words, while mtDNA is misleadingly similar in size and complexity to a large bacterial plasmid today, it could only become so through the reductive evolution of the full bacterial genome, such that steady gene loss over many generations was continuously compensated for, within the host cell as a whole, by equally gradual improvements in intracellular trafficking, evolving over many generations.

Thus there are two overwhelming reasons for why prokaryotes remain prokaryotic. First, scaling up to a eukaryotic volume requires scaling up the number of genomes accordingly, which undercuts the energy available per gene by five orders of magnitude. Second, multiple genomes are necessary to retain control of oxidative phosphorylation over a wide area of bioenergetic membranes. Literally, only endosymbiosis solves both problems, because cytoplasmic inheritance enables the evolutionary loss or transfer of genes and DNA to the nucleus, which in turn permits the evolution of intracellular trafficking systems at no net energetic cost, while leaving in place the core genomes necessary for respiration across a wide area of membranes.

The fact that endosymbiosis is strictly necessary to evolve beyond the prokaryotic way of life, but is exceptional (if still documented) in prokaryotes, goes some way to explaining why eukaryotes only arose once in four billion years of evolution. But the next step--converting an endosymbiont into an organelle--was equally challenging, as attested by the absence of evolutionary intermediates.

### Endosymbiotic gene transfer, mutagenesis and the origin of sex

Besides steadily transforming the energy availability per gene, mitochondria influenced their host cells in other critically important ways, notably by bombarding the host cell genome with DNA: not only genes but also genetic parasites such as spliceosomal introns [59,111].

Prokaryotes assimilate DNA from their environment by lateral gene transfer. This is especially true when DNA derives from their own internal environment [112]. Lysis of endosymbionts releases DNA into the cytosol of the host cell, which can be integrated into the host cell chromosome by standard non-homologous recombination. If there is more than one endosymbiont, DNA is transferred via a gene ratchet from the endosymbionts to the host cell (if the host cell lyses, the endosymbionts die along with their host, so DNA only rarely flows the other way). The reality of endosymbiotic gene flow is testified by numts (nuclear mitochondrial sequences) [113] and nupts (nuclear plastid sequences) [114], which accumulate in the nuclear chromosomes despite physical obstacles, such as the nuclear membrane, chromatin packing and cell-cycle check-points. Presumably, in the absence of a nucleus in a prokaryotic host cell, such mutagenic insertions would have been more common and more serious.

Lateral transfers to the nucleus also occur with other eukaryotic endosymbionts such as *Wolbachia*, with repeated transfers to the genomes of insects and nematodes, ranging from nearly the entire Wolbachia genome (> 1 Mb) to shorter insertions [115,116]. Lateral transfers frequently assimilate into genes, and numts are known to cause *de novo *genetic diseases [113] and potentially contribute to ageing [117,118]. Most data on numt and nupt accumulation refer to germline acquisitions over evolutionary time, but the actual mutation rate can be much higher during the chronological lifespan in yeast and mice [117,118]. Such mutations, of course, would mostly not have been in the form of nucleotide substitutions but rather aberrant recombinations: insertions, deletions, duplications, transpositions and other rearrangements. Aberrant recombinations frequently cause cytoplasmic male sterility in plants today [119], and are also found in *Wolbachia *endosymbionts, some of which have a remarkably high density of mobile group II introns and other transposable elements [120].

The fact that around 75% of eukaryotic genes with prokaryotic sequence similarties are related to bacteria rather than archaea [121] hints at the possible scale of such an early endosymbiotic DNA bombardment, albeit not all of these genes are necessarily derived from mitochondria. As noted above, the very existence of mitochondria abolished the selective penalty for accumulating genes and DNA in the nucleus, so endosymbiotic gene transfer alone might have driven the increase in nuclear genome size to some extent. However, three factors suggest that simple endosymbiotic gene transfer was not the most important driver of genome size. First, as argued earlier, the absence of evolutionary intermediates suggests the intermediates were not stable, implying a mutagenic process. Second, the common ancestry of many large gene families in eukaryotes attests to the importance of gene and whole genome duplications in early eukaryotic evolution [122,123]. The last eukaryotic common ancestor (LECA) had already increased its genetic repertoire by some 3000 novel gene families [122,123]. And third, the deep conservation of intron position suggests that many introns were already present in LECA [1,59,111]. Given the likely origins of eukaryotic spliceosomal introns as mobile group II introns deriving from the bacterial endosymbiont [124-126], it is likely that the archaeal host cell was subject to an early bombardment of introns that proliferated throughout the host cell genome, before decaying [111,122]. This heavy intron bombardment has been argued, compellingly, to have driven the evolution of the nucleus as a way of separating the slow splicing of intronal RNA after transcription from the somewhat faster process of ribosomal translation, thereby avoiding the synthesis of aberrant proteins [127].

Some suggest that such an early proliferation of introns would only have been possible in a sexually reproducing host cell [128], despite examples to the contrary [120,129,130]. In fact it is more likely to be exactly the other way round: the high mutation rate exerted by intron replication and gene transfer might have driven the evolution of sex, or at least cell fusions and genome doublings, very early in eukaryotic evolution, potentially aiding intron spread even further. Introns induce mutations in part by inserting themselves into functional genes, but also, notably, through aberrant recombinations [131], which have the potential to break circular bacterial chromosomes into straight chromosomes [111]. In providing the ribozyme machinery requisitioned by telomerase enzymes, spliceosomal introns offered up at least part of the solution to the problem of straight chromosomes [111]. Even so, the host cell must have had nearly insurmountable problems in getting through its life cycle, faced as it was by a high mutation rate from the bombardment of genes and introns, variable numbers of now straight chromosomes, and presumably, an inability to divide by the standard archaeal chromosomal segregation.

One conceivable way in which the host cell might have segregated its straight chromosomes would have been by utilising plasmid segregation machinery [132], and it is notable that some prokaryotes can segregate large plasmids on TubZ microtubules, functionally equivalent to the microtubules in the eukaryotic spindle [133-135]. The dynamism of cytoskeletal components, both actin and tubulin, could have been promoted by the loss of genes from mitochondria, as discussed earlier, permitting the *de novo *synthesis and assembly of monomers, and indeed motor proteins, at no net energetic cost. Notably, the early evolution of a dynamic cytoskeleton would have permitted the loss of the prokaryotic cell wall (as presumably happened before LECA, given the lack of homology in the walls of fungi, algae and protists). The possibility that phagocytosis evolved independently on three separate occasions early in eukaryotic evolution does indeed suggest an early evolution of a dynamic cytoskeleton [136] but still *after *the acquisition of the mitochondrial endosymbiont. Likewise, the internalization of oxidative phosphorylation in mitochondria may have reduced the need for a cell wall [137]. Whatever the reasons, the loss of the cell wall would presumably have facilitated cell fusions; and it is plausible, if unsubstantiated, that such fusions might have been manipulated by mitochondria (which could only invade a new host via cell fusion) [138]. By masking mutations and facilitating the spread of mitochondria, cell fusions would have benefited both the host cell and the endosymbiont.

Cell fusions and genome doublings, as in allopolyploid speciation in many plants today [139], could potentially explain the origin of the eukaryotic cell cycle, and work on a mathematical model here is ongoing. The salient point is that a high mutation rate combined with the expansion of bioenergetic and genomic capacity over several orders of magnitude permitted a radical solution not available to prokaryotes: cell fusion masked mutations in genes, while doubling the genome enabled cell division despite variable numbers of chromosomes. This model could also potentially explain why eukaryotes have accumulated the entire basal set of eukaryotic traits, thus beginning to explain the evolutionary void between prokaryotes and eukaryotes.

Regardless of how early eukaryotes escaped from their predicament, it is plain that the problems faced by a prokaryotic host cell with bacterial endosymbionts are serious, if not irreconcilable, and go a long way towards explaining why there are no surviving evolutionary intermediates between prokaryotes and eukaryotes. It is easy to see why all the intermediates should all have fallen extinct; harder to model a path through the thicket of problems. On the other hand, presumably there must be an evolutionary explanation for why eukaryotes have deeply conserved intron positions, straight chromosomes, telomeres, the nucleus, large genomes, non-coding DNA, a microtubule spindle, cell fusions, multiple genome duplications, large gene families and meiotic (reciprocal) sex. Remarkably, the hypothesis developed here predicts all these traits as strikingly plausible consequences of a cell within a cell.

## Conclusions

All complex life on Earth is eukaryotic, and eukaryotes arose just once in four billion years of evolution, via a singular endosymbiosis between prokaryotes. The acquisition of bacterial endosymbionts by a prokaryotic (archaeal) host cell is an extremely rare occurrence, albeit there are known examples in walled prokaryotes in the absence of phagocytosis [32,33]. Cytoplasmic inheritance of endosymbionts is the only mechanism able to solve the scaling problems faced by prokaryotes expanded to eukaryotic genome size and cell volume. Prokaryotes can be expanded to eukaryotic volume via extreme polyploidy [23], but cytoplasmic inheritance is strictly necessary for energetic expansion, as it alone enables the loss of the vast majority of endosymbiont genes, while retaining the few genes needed to maintain control over oxidative phosphorylation across a wide membrane area [34]. This extreme genomic asymmetry is in fact diagnostic of eukaryotes, and enabled the expansion of both bioenergetic membrane surface area and genome size over several orders of magnitude.

This bioenergetic expansion was permissive, not prescriptive. The actual increase in eukaryotic genome size was mainly driven by the bombardment of genes and introns from endosymbionts, which caused a high mutation rate, breaking the circular prokaryotic chromosomes into straight eukaryotic chromosomes [111]. Cell fusions and genome doublings were made feasible by a combination of the loss of the cell wall (no longer needed in the presence of a dynamic intracellular cytoskeleton) and relief from the heavy prokaryotic selection pressure to lose genes and DNA. By masking mutations and enabling a functional cell cycle, cell fusions and genome doublings laid the foundations of the eukaryotic sexual cell cycle. The major side effect of the protosexual cell cycle was the accumulation of all basal eukaryotic traits in a single population, giving rise to the LECA, utterly different in its genetic and morphological makeup to all known prokaryotes. The perspective developed here explains why the eukaryotic cell arose just once in 4 billion years of evolution; why there are no surviving evolutionary intermediates between prokaryotes and eukaryotes; why eukaryotes are morphologically complex and share many common traits; and why reciprocal sex arose in eukaryotes but not in prokaryotes. All these unexplained features of the prokaryote-to-eukaryote transition unfold in effortless inference as highly plausible consequences of an endosymbiosis between prokaryotes.

## Reviewers' Comments

### Reviewer 1

Eugene V. Koonin, National Center for Biotechnology Information (NCBI)

This is a very interesting, provocative paper that perfectly fits the "Beyond the TOL" series as it tackles in earnest an evolutionary process that was essential for a major evolutionary transition, the origin of eukaryotes, but had nothing to do with TOL, namely endosymbiosis and the ensuing extensive gene flow from the mitochondrial to the nuclear genome. Lane brings a welcome energetic perspective to this issue and uses specific numbers to address it. This is an excellent approach. Having said this, I have a variety of problems, questions and misgivings which I list and discuss below in the order they appear in the article rather than in the order of perceived importance. A summary of key points is given at the end of the review.

### Author's response

Thank you. I address the problems, questions and misgivings below and in the text as appropriate

The first section is entitled "The origin of the eukaryotic cell was a unique event". I am not at all convinced by the argument here. There is a big difference here with the statement, in the first sentence: "There is little doubt that all known eukaryotic cells share a common ancestor that evolved only once in four billion years of evolution". I believe there is not "little" but no doubt whatsoever that all extant eukaryotic cells had a common ancestor. The evidence is overwhelming (parenthetically, the second part of the quoted sentence is at face value oxymoronic: if there was a common ancestor, certainly it evolved once). A single origin of ALL eukaryotic cells is a different matter. I am not compelled by the argument against extinction of many lineages of protoeukaryotes. As pointed out in this article and many others, the early stages of eukaryogenesis were a trying time for the chimaeric organism(s), and there might have been quite a few botched trials. Unfortunately, we indeed do not have a good way to find out, so to me the only argument for the uniqueness of eukaryogenesis is Occam's razor.

### Author's response

*I agree that there is "no doubt" that eukaryotes share a common ancestor, at least in my own mind; nonetheless, without more formal proof, 'no doubt' seems too strong. I mention two other possible mechanisms of inheritance by which eukaryotes could feasibly share common traits, and note that they are far less likely than common ancestry to explain the observations. But if Occam's razor is the best proof then the case must be considered formally unproved, as I indicate*.

*We seem to agree about extinction. My argument is against the facile invocation of extinction to account for the absence of true archezoa, which is to say, against the former existence of a primitive nucleated phagocyte as host cell to the mitochondrial endosymbiont (i.e. eukaryogenesis proceeds largely in a non-chimeric cell). I have clarified this in the manuscript. The fact that morphologically simple eukaryotes, formerly known as archezoa, actually do exist, is the critical point, recognized incidentally in the review by Ford Doolittle. Plainly these primitive eukaryotes are not driven to extinction by competition from more complex eukaryotes. The niche for morphologically simple eukaryotes, lacking mitochondria, ER and many other standard eukaryotic traits, is not only viable, but is filled with morphologically simple eukaryotes that have not been outcompeted to extinction by more complex eukaryotes. Yet every single one of these 1000 or more species arose by reductive evolution from more complex ancestors. On purely statistical grounds this is very unlikely, as I argue. It is more likely that the dice was loaded in some way, such that reductive evolution from a more complex ancestor is altogether more likely than complexification (ugly word--is there a better one?) of prokaryotic ancestors*.

*Now if that is true--there are ecological intermediates but no true evolutionary intermediates--then it follows that there are a lot of extinct true evolutionary intermediates between prokaryotes and the last eukaryotic common ancestor. Koonin does not disagree with this perspective; on the contrary, he writes that eukaryogenesis was a 'trying time for the chimeric organism'. I couldn't agree more. My whole point is that there were specific reasons for it being a trying time, not least Koonin's own 'intron catastrophe', which I discuss at some length later in the paper. So what I object to is the glib assertion of 'extinction' of all true archezoa through competition, not the fact that eukaryogenesis was a trying time. My whole paper is really an exploration of why prokaryotes do not tend to become eukaryotic, and why, of all chimeric prokaryotes that must have existed in the past (we know of two examples today which are patently not eukaryotic), only one line survived. This gave rise to a surprisingly complex last eukaryotic common ancestor, and only after that to the simpler ecological intermediates that we know, via reductive evolution*.

"What prokaryotes lack is the characteristic eukaryotic accumulation of all of these traits at once, bound up in massive, wasteful genomes [34]."

I think one should tread carefully here and try to be explicit. First of all, these traits are combined not so much in genomes, but in eukaryotic cells themselves (obviously, the respective genes are combined in genomes). That's semantics, though. More importantly, a great many eukaryotic genomes are not that large and only minimally more wasteful (in terms of the size--and hence the replication cost--of the genome itself) than the (relatively) large genomes of prokaryotes. The really dramatic wastefulness is seen only in a few lineages, primarily multicellular organisms (above all, vertebrates) as well as some free-living amoebae. Many unicellular eukaryotes have compact genomes, only slightly less compact than prokaryotes. Have all compact eukaryotic genomes been secondarily streamlined, some quite dramatically? This is a distinct possibility (see for example [59]) but it would be best to discuss it explicitly.

### Author's reply

*This is a fair point, and I have revised the manuscript in several places accordingly, to give range values. However, I do not agree that profligate genomes are restricted to multicellular organisms, and mostly plants and vertebrates. According to data from Gáspár Jékely and Tom Cavalier-Smith (personal communication, collated from references 62-67) the mean genome size of microsporidia is around 10 Mb, and fungi around 40 Mb, in the range mentioned by Koonin. However, fungal genome sizes range up to 1000 Mb, while the mean for mitochondriate phagotrophs is about 700 Mb, ranging up to 10,000 Mb. The mean genome sizes of algae are larger again, at around 3000 Mb for Cryptophyceae and Dinoflagellates--the value discussed in the paper--ranging up to 10,000 and 100,000 Mb respectively*.

*My point is not that the acquisition of mitochondria determines genome size, but that it released an energetic constraint faced by all prokaryotes (their largest genome size being barely more than 10 Mb). Almost certainly the actual genome sizes of these modern groups is set by factors such as cell volume, nuclear volume and lifestyle; but in terms of lifestyle it is striking that Chlorophyta and cyanobacteria share an equivalent lifestyle--oxygenic photosynthesis--yet the mean genome size of Chlorophyta is about 500 Mb, compared with less than 10 Mb for cyanobacteria. Why the 50-fold gap? My answer is that cyanobacteria are constrained in genome size for energetic reasons but that Chlorophyta are not, and so have ultimately adapted to a larger cell volume and genome size, set by other constraints no doubt, but permitted by mitochondria*.

"...there was no successful speciation across the prokaryote-eukaryote transition, despite the great evolutionary distance. Over this transition, the tree of life is neither a branching tree nor a reticular network, but an unbranching trunk (Figure 2)."

I have difficulty understanding what "speciation across ...transition" means but I assume that here again the monophyly of all extant eukaryotes is the point. This is true, regardless of how much extinction of protoeukaryotes might have taken place, but is it surprising? I think only if one takes a very specific view of the biological world as two "empires", prokaryotes and eukaryotes, then the uniqueness of the connecting trunk appears striking. However, if one simply views eukaryotes as a monophyletic group (clade), then the existence of unique trunk (same as root) is not that remarkable.

### Author's response

By transition, I mean the evolutionary gap between a prokaryote within a prokaryote--what I would take to be an alpha-proteobacterium inside an archaeon, without a nucleus or other eukaryotic traits--and the last eukaryotic common ancestor, which had a nucleus, straight chromosomes, introns and exons, nuclear pore complexes, mitosis and meiosis, mitochondria, ER, dynamic cytoskeleton, motor proteins, etc, etc. This is a long evolutionary distance by any account. It is not merely the root of the eukaryotic "tree" but a very long branch indeed (which I describe as a trunk because it is beneath the base of the eukaryotic tree). Unlike long-branch artefacts, this distance is not an artefact, because the evolutionary distance is real. The question then becomes, why do we not see speciation in this long evolutionary journey between a cell within a cell and LECA? Why do we not see some eukaryotes without mitochondria, others without meiosis, some without a nucleus, others without an endoplasmic reticulum? Of course we do see eukaryotes without some of these traits, and we used to call them archezoa. If the archezoa were "real", then they would represent early branches of the eukaryotic tree, i.e. speciation across the prokaryote-eukaryote divide. But it now turns out that the archezoa are not true evolutionary intermediates, but evolved from a common ancestor that did have all these traits. So the question has even more force: why do we not see any true intermediates? Rephrase: why do we not see any early branching species? As I note above, the standard glib answer is that they were all outcompeted to extinction, but the existence of plentiful ecological intermediates--the archezoa--questions that conclusion. What we see instead, as Koonin himself points out, is something more like a big bang: no early branching at all. Why not?

*The answer that I give might be wrong but at least it gets at the question. Ironically, my answer here is no more than the answer that Koonin himself gives, i.e. we do not see intermediates because the intron catastrophe, and other nearly intractable consequences of a cell within a cell, made early eukaryogenesis a trying time. I go further to suggest that it was so trying that the evolution of sex was the only way out. Unlike bacteria, cell fusion and genome doublings were permitted by the lifting of energetic constraints, and allowed escape from an unprecedentedly high mutation rate--caused by a heavy bombardment of endosymbiotic DNA--but had the side effect of accumulating traits in a single population. Traits could accumulate because there was no heavy selection pressure to lose them on energetic grounds. I readily admit that this is speculation, but so is any hypothesis. The real question is, is it testable? Not easily, obviously, but potentially so. I am working with colleagues at UCL on a mathematical model which I hope might give some insights, especially into the question of mutation rates that can be mitigated by lateral gene transfer versus meiotic sex. So this paper sketches the outlines of a testable hypothesis*.

"Spatial structuring of stable populations should have led to divergence and speciation, at least some of which ought to have permanently occupied the Archezoan niche."

What is that niche? I am afraid I do not really understand the reasoning here.

### Author's response

*I have made this clearer in the article, also in response to Bill Martin's query. The archezoan niche is the niche that is occupied by morphologically simple unicellular eukaryotes, the ones that we used to call archezoa. In other words, there is an ecological niche between prokaryotes and more complex eukaryotes which is occupied by 'primitive' eukaryotes. If the transition from prokaryotes to eukaryotes was a walk in the park, then archezoa would have been real evolutionary intermediates. It wasn't a walk in the park. The archezoa are real ecological intermediates, so there is nothing wrong with the niche itself. Simple eukaryotes without mitochondria do perfectly well--they have not been outcompeted to extinction. My point is that if there was nothing wrong with the niche then there must have been something wrong with the intermediates--for example they comprised a highly unstable small population that never managed to thrive or do anything other than hang on for dear life while mating (fusing cells) to survive, and incidentally accumulating traits. Finally, these early eukaryotes achieved some kind of stability, by which time they were already quite complex and close to LECA. There were no true archezoa because the early eukaryotes themselves were unstable, not the niche. The big bang of eukaryotic evolution (the very short branches between the eukaryotic supergroups) occurred as soon as LECA became stable enough to thrive, diverge, and invade different niches. This was, quite literally, the origin of species*.

"An 'average protozoan' has 3000 Mb of DNA giving it a power of 0.76 pW Mb"

I am not sure that this is a fair value for the mean size of a protozoan genome. I realize that the number comes from the comprehensive review by Gregory [63] but I wonder about the contribution of inaccurate measurements as well as polyploidy to these estimates. In any case, the important point is that the genome size range of 10-30 Mb is densely populated among protists and fungi (see for example [140], and by no means are all these organisms parasites with degraded genomes. In principle, the most relevant number here would be the genome size of LECA. Certainly, we do not have that number but I strongly doubt it was in the gigabase range. Accordingly, when estimated per megabase of the nuclear genome, the metabolic power would be three orders of magnitude greater than in prokaryotes. The estimate of the total mitochondrial genome size in the article seems to be correct, so when it comes to the sum total, the estimates may be not that far off. Nevertheless, the above requires attention.

### Author's response

*I agree with all of this, and have modified the text accordingly. I too wonder about the contribution of polyploidy. Plainly this is greater than has been appreciated. But the existence of polyploidy does not undermine the arguments set forth here and in my Nature paper with Bill Martin *[34]*in any way; on the contrary, it is central to it. Some eukaryotes are highly polyploid; for example Paramecium, which can have 800-1000 copies of the macronucleus, a serious genomic burden. In this context it is notable that almost all introns and intergenic material is removed from the macronuclei, paring them down to a minimum. Paramecium nonetheless has 40,000 genes, incidentally somewhat more than most vertebrates. In terms of energy per gene, it can only just support this genome size, on practically bacterial rations per gene; so it is interesting that the genome in macronuclei (not micronuclei) is streamlined in essentially bacterial fashion. There is no reason to suppose that this streamlining has anything to do with replication speed (see below)*.

*Certainly there are issues about the accuracy of genome size estimates. For many years the genome size of Amoeba dubya was cited as the largest known eukaryotic genome, at an extraordinary 670,000 Mb, memorably 200 times the size of the human genome. More careful studies have revised this number down to around 100,000 Mb. Replicating this amount of DNA certainly has an energetic cost (see below), especially the portion of it that codes for proteins, and in that respect it doesn't matter whether it is diploid, triploid (as has been claimed) or massively polyploid. The most serious cost is the cost of protein synthesis--the number of ribosomes--which affects polyploids as much as haploids*.

*I agree that the most relevant number here would be the genome size of LECA; but even then we could not be certain what the mitochondrial genome size was at that point in eukaryotic evolution, so there is necessarily a sliding scale. I have added range values to the paper (some of which were also given, along with some specific examples, in the Nature paper *[34]*). The main point, which persists through all of this, is that prokaryotes face tight energetic constraints, which are not solved by giant size and polyploidy (on the contrary energy per gene is less than in E. coli), but which are released by mitochondria in eukaryotes, allowing essentially a free expansion in size up to the transport limits of single cells, or conversely, a paring down via reductive evolution even to the point of competing with prokaryotes on prokaryotic terms (ie fast replication, streamlined genomes, and restricted energy per gene). Thus again, energetics permits expansion of genome size but does not prescribe it*.

*If, as Koonin suggests, the genome size of LECA was in the order of tens to hundreds of Mb, then the arguments of the paper are only strengthened, as the distinction between prokaryotes and eukaryotes simply widens, to three orders of magnitude, as Koonin observes. I note this is the paper*.

The per gene estimates are not very convincing (and the numbers seem not quite accurate). An average bacterium hardly has about 5,000 genes. The current distribution is bimodal, and the mean will come closer to 2,000-3,000 genes. More importantly, however, there are many bacteria with ~10,000 genes (e.g. [141]), not many fewer than, say, *Drosophila*, a rather complex eukaryote by any account. I do not find it plausible that, say, doubling the number of genes in bacteria is strictly prohibited due to energetic constraints. In more general terms, I am unsure that metabolic power per gene is in itself particularly important. So I question this conclusion "The lower limit is set by the number of genes necessary for a free-living existence; the upper limit by energetic constraints." We do not know what is the dominant factor that determines the upper bound on the number of genes in prokaryotes and whether it even makes sense to speak of such a factor. However, I am rather confident that energy limitation is not it. A better candidate at least seems to be the "bureaucratic ceiling" hypothesis maintaining that the quadratic scaling of the number of regulators with genome size at some point renders further genome growth unsustainable [141,142]. Metabolic power per gene may be an important quantity but in terms of increasing the number of genes but rather in terms of the cost of expression in the large eukaryotic cells.

### Author's response

*A mean of 5000 genes is just over the genome size for E. coli, which is hardly untypical. I have given range values in the paper for both 2,500 and 10,000 genes. Neither value alters the conclusions, as in any case noted by Koonin*.

*The larger question here is whether or not energetic constraints determine the upper bound on the number of genes in prokaryotes. Of course my argument that they do is a hypothesis. It is supported by the fact that the energy-per-gene shrinks in larger bacteria, and is miniscule in giant bacteria, even though they have small haploid genome sizes (typically in the range of 3-4 Mb with little more than 3000 genes). These small haploid genome sizes are predicted on bioenergetic grounds--giant bacteria simply do not have the energy to support large genomes, despite (or rather, because of) the fact that they sustain massive amounts of DNA overall*.

*Larger cells do not have more energy per gene expressed; on the contrary, they have less. Three facts are important: (i) protein synthesis accounts for 75-90% of the ATP budget of growing bacteria, so there is a near-linear relationship between gene number and ATP expense; (ii) initiation of bacterial replication depends on the ATP/ADP ratio (e.g. via the binding of multiple ATP-bound DnaA proteins to OriC, the replication origin), therefore replication depends on the rate of ATP synthesis in relation to protein synthesis; and (iii) larger cells face surface-area-to-volume constraints in the rate of ATP synthesis relative to protein synthesis. Given these three factors, it is inevitable that larger cells will be constrained in genome size at some point, unless they can internalize respiration. Obviously they cannot internalize respiration on anything like the eukaryotic scale (they fall 3-5 orders of magnitude short--that is an empirical observation). So where does this limit lie? I would suggest that it lies around 10,000 genes with 10 Mb of DNA. This statement is a testable hypothesis, and is borne out by known data*.

*What about the "bureaucratic ceiling hypothesis" favoured by Koonin? Why should the quadratic scaling of regulators limit prokaryotes but not eukaryotes? From an energetic point of view, the answer is simple: bacteria have a limit on the total number of genes, and without additional genes to regulate, what is the point of having more regulators? None. Even a small cost without an advantage will be penalized by selection for replication speed. It has been argued that the difference between eukaryotes and prokaryotes lies in regulation, and that eukaryotes don't necessarily accrue massive energetic costs because transcription factors can be produced at low copy number, or as micro RNAs, eliminating the costs of protein synthesis altogether. We addressed this view in detail in our Nature paper *[34]*, so I have not dealt with it further here. The observed fact is that protein synthesis does indeed scale with gene number in eukaryotes, as can be seen from a simple consideration of ribosome numbers. E. coli, for example, has up to 13,000 ribosomes, whereas a human liver cell has 13 million on the rough endoplasmic reticulum alone -- 1,000 to 10,000-fold more, entirely in keeping with the arguments set forth here. Eukaryotes have more regulators because they have a lot more to regulate. The proteins that they regulate make up the morphological complexity of the eukaryotic cell, which is and has always been the most striking difference between eukaryotes and prokaryotes*.

*Incidentally, a bimodal distribution of genome size in bacteria is predicted on energetic grounds. Very small cells will be favoured in terms of replication speed, but must support a minimum number of genes for a free-living lifestyle. The smaller the cell, the smaller the absolute plasma membrane surface area for chemiosmotic coupling, which constrains energy per gene for very small cell sizes because the genome size is necessarily quite large in relation to surface area. As cells become larger, they presumably reach some kind of energetic optima in the region of 2,500-5000 genes. Much larger genomes, up to 10,000 genes, are not generally favoured, except in cells that have complex internal membranes, such as cyanobacteria and nitrifying bacteria. These complex bacteria are under less heavy selection pressure for replication speed (I assume because they draw on resources unavailable to other bacteria), but are still far less complex than quite mundane unicellular algae like Euglena. Why the gap? Many cyanobacteria with complex internal membranes are also polyploid, which permits a larger surface area of bioenenergetic membrane, as in giant bacteria like Epulopiscium and Thiomargarita, but with all the costs and limitations discussed for them. I am not aware of systematic studies of metabolic rate in large cyanobacteria, but such studies could give invaluable insights into the energetic limitations of large bacterial genomes*.

"It is notable that eukaryotes support, on average, around 500 times more DNA than prokaryotes but only four times as many genes."

This is more or less correct about genes but dubious at best when it comes to DNA: only some animals, polyploid plants and a few protist lineages have genomes that big.

### Author's response

I have already addressed this above

"Bacteria therefore maintain high gene density, around 500-1000 genes per Mb, and do so by eliminating intergenic and intragenic material [72-74], including regulatory elements and microRNAs, by organising genes into operons, and by restricting the median length of proteins [75]."

Bacteria do show a trend for genome streamlining. However, I once again doubt that the energetic cost is the defining factor here. Bacteria are after all highly efficient organisms that are capable of producing proteins at an extremely high rate and rapidly divide. At least under favorable conditions, they do not experience any shortage of energy. Energetic considerations might come into play as an explanation for the contraction of proteins but when it comes to intergenic regions, I suspect that speed of replication is more important (in bacterial populations with their large characteristic effective population size, even a small decrease in the speed of replication is "seen" by purifying selection). On a different issue arising from these same passages: It seems somewhat disingenuous to speak of genome streamlining resulting from elimination of regulatory elements and microRNAs. The implication here seems to be that ancestral bacteria had those, and this is most likely not the case. Rather, any genome expansion in bacteria is tightly controlled by selection, so bacteria have evolved these features only to a limited extent.

### Author's response

*I agree that replication speed is more likely to be responsible for the elimination of small amounts of intergenic and intragenic material in bacteria, subject to the provisos mentioned earlier (that there is no point in having additional regulatory elements if there is nothing for them to regulate)*.

*However, DNA replication accounts for about 2% of the ATP budget in growing microbes. Increasing the DNA content by 10-fold would increase the cellular energy budget by about 20%, presumably a relatively trivial cost, at least in comparison with increasing gene number by 10-fold (which would increase energy budget by nearly 10-fold). But increasing genome size to a eukaryotic mean of 3000 Mb (see earlier discussion to justify this value) would increase energy demand 12-fold, not a trivial consideration. Unicellular algae and protozoa have genome sizes ranging up to 100,000 Mb so the cost would be even greater. Even expansion to a relatively small eukaryotic genome size of 100 Mb would mean that 40% of the cell's energy budget would need to be spent on totally pointless DNA replication, which would undoubtedly be penalized by selection, as noted by Koonin*.

*There is a broader point here which brings me to Koonin's second point. I am not for a moment suggesting that bacteria face any ATP limitation. Plainly they do not. What I am suggesting is that they would face an ATP limitation if they stopped being bacteria and evolved in the direction of being eukaryotic--larger cell volume, larger genome size, more proteins, more regulation. Then they would undoubtedly suffer an ATP shortage for exactly the reasons I discuss in this paper. The fact that we do not see bacteria evolving into eukaryotes is perfectly explained by the fact that prokaryotes lie in a deep canyon in the energy landscape, with high energetic barriers preventing their escape. Bacteria have remained bacteria precisely because as bacteria they do not experience any shortage of energy. Eukaryotes alone tunneled through the walls of this energetic canyon, because eukaryotes alone have mitochondria, which always retain genes, which enable local control of chemiosmotic coupling and so give them a freely expandable source of ATP. This eliminates the energetic barrier to larger cell volume, large genome size, etc. This in itself does not explain why eukaryotes evolved as they did, it merely removes the energetic barrier to evolution; but it does explain why prokaryotes do not tend to become eukaryotic*.

"For example, one of the smallest anaerobic phagotrophs, *Entamoeba histolytica *has nearly 1000 genes [78]--more than the largest known bacterial genome"

*E. histolytica *has nearly 10,000 genes (not 1000) but this is fewer than the largest known bacterial genomes (Myxococci).

### Author's response

*An unfortunate typo. It is in the same order as the largest known bacterial genomes. I have addressed this point in more detail in response to Mark van der Giezen's comments*.

Here is a very general point: "Eukaryotes have 1000-5000-fold more energy to burn per gene *because *they are larger."

I think this turns the issue on its head: *because *eukaryotic cells are indeed much larger, they need to generate much more energy per gene, to produce the proteins required for the maintenance of those large cells.

### Author's response

*I have modified the wording. The point, as developed above, is that bacteria do not become larger because if they do they have less, not more, energy per gene. Certainly eukaryotes need to generate much more energy per gene because they are larger, but then so would bacteria. Bacteria are not larger because they cannot generate a lot more energy per gene*.

"The issue of scaling" and the next two sections: Here I think convincing (if not exactly compelling as I discuss below) arguments on the inevitability of endosymbiosis for the evolution of complex cells are starting to appear although I am not certain that they have been developed to the extent necessary. I still do not understand some numbers like "The deficit of prokaryotes relative to eukaryotes is then apparently reduced to a mere 8-fold..."-where does the number 8 come from? Regardless, though, I think the important numbers are straightforward: "If the rate of protein synthesis is unchanged, but the surface area of plasma membrane is increased 625-fold, then ATP synthesis per unit area must fall by 625-fold (not even allowing for inefficiencies in intracellular transport). It is impossible for a bacterium to step up the rates of transcription and translation by 625-fold from a single genome; the only reasonable solution to scaling up on such a scale would be to scale up the total number of genomes accordingly--most reasonably, assuming that streamlined bacteria already approach the limits of efficiency, by a factor of 625-fold". Then, this argument I believe is important: "Rather than extreme polyploidy, eukaryotes exhibit extreme genomic asymmetry". Indeed, such asymmetry sharply divides eukaryotes from any giant bacteria known. Why this is possible in eukaryotes is clear--gene transfer from mitochondria to the nuclei and even more importantly, elimination of many bacterial genes. Indeed, if all endosymbiont genes except for a handful remaining in the mitochondrial genomes, were transferred to the nuclei, then expressed and the proteins transported back to the mitochondria, there would have been no energetic gain as the same amount of protein would have to be produced anyway. The trick (not mentioned in the paper) is that only a few hundred genes from the original bacterial symbiont are transferred to the nucleus whereas the rest are lost irreversibly, being superfluous in the intracellular environment. What is less clear to me, is why the giant bacteria would have to rely on extreme polyploidization as the only route to efficient energetics (a route that at the end is a blind alley). If one speaks of "reasonable solutions", why not evolving a high ploidy plasmid that would carry all genes required for the formation of energy-generating complexes? I do not think that this claim "That makes extreme polyploidy fundamentally inflexible--unevolvable--and all the copies of an identical genome must remain exactly that--copies of an identical genome, with no potential to lose genes" is very strong: there is such potential leading to a plasmid (there is no impenetrable barrier between prokaryotic "chromosomes" and "megaplasmids"). Later on, Lane brings up this possibility and concludes that "...the cell would have plenty of ATP, but would still be unable to distribute any other proteins or substrates about the cell in the absence of highly evolved transport networks. It would fail and die." This is a sensible conclusion. There is simply no way to make a large cell functional (without an inert internal space) other than developing elaborate intracellular compartmentalization, that is ...becoming a eukaryote (or at least "eukaryote-like").

### Author's comments

*The 8-fold reduction: The mean metabolic rate of an average protozoan, as cited in the paper, is 2,286 pW. The mean for bacteria is 0.49 pW. If the bacterium were expanded by 25-fold in linear dimension, giving it an equivalent volume to a protozoan, the surface area would increase 625-fold. Assuming a fixed rate of ATP synthesis per unit area, the metabolic rate would be 0.49 × 625 = 306 pW. This is 7.5 times less than 2,286 pW (rounded up to 8-fold)*.

*I agree that if all the genes transferred to the nucleus were expressed, there would be no energetic gain. It is not true that I do not make this point. I based two paragraphs of calculations on the energy savings made if a proportion (initially 5%) of endosymbiont genes were not necessary in the context of an endosymbiosis, and so were lost altogether (paragraphs described as 'spectacular' by Bill Martin). The calculation showed that if this loss occurred in a cell equivalent to Epulopiscium, the energy savings would be sufficient to fuel the de novo synthesis and self-assembly of 800 microns of actin filaments every second. Ultimately the energy savings stem from the specialization of the endosymbiont as an energy-transducing organelle, as emphasized by Bill Martin in his review (and addressed there). It is in fact misleading to talk of energy savings, because they are spent on other things, such as the evolution of dynamic transport networks, at no net energetic cost. Transport networks enable other savings, because they provide the endosymbionts with more of what they need, allowing the endosymbionts to lose more genes. Again the energy savings are virtual, and are spent on the evolution of other proteins, such as motor proteins (or whatever)*.

*I think it is likely that far more than a few hundred genes were transferred from the mitochondria to the host cell nucleoid. The host cell nucleoid was surely bombarded with DNA. The point is that most of this DNA was no longer necessary for its original purpose. It became pseudogenes, and new gene families, the basis of variation and evolution. It is correct to say that not duplicating functions was key, the specialization of the endosymbiont as an energy-transducing organelle. The critical point, which I make clear, is that the fundamental difference between eukaryotes and prokaryotes is not the total amount of DNA that can be sustained (that is quite similar in eukaryotes and giant prokaryotes) or the cell volume (again that can be similar) but the genomic asymmetry, in which tiny specialized mitochondrial genomes support a giant host cell genome. That giant host cell genome is not filled with repetitive copies of the same gene making the same protein, but is free to vary, a point I make specifically in Figure *3.

*Finally: plasmids. It is an empirical observation that all known giant bacteria have multiple nucleoids rather than plasmids associated with their plasma membrane. In the Nature paper *[34]*we addressed the issues with plasmids in some detail and I have not reiterated that here. Briefly, bacteria tend to have either multiple copies of small plasmids, which segregate randomly at cell division, or a few copies of giant plasmids, which segregate like chromosomes on a protein scaffold. A plasmid containing all the genes necessary for oxidative phosphorylation is not unthinkable, but would need to contain several score genes, and so would be a giant plasmid. How these genes would come to sit together on a single plasmid is a question we can put to one side. Assuming that such a plasmid existed, it would need to be present in potentially hundreds or thousands of copies, and segregated on a protein scaffold every cell division. That goes against the bacterial grain. Worse, these multiple giant plasmids would need to associate closely with the plasma membrane at roughly equal distances, each and every generation--again not a typical bacterial trait. These reasons seemed strong rather than compelling. It is still not unthinkable that a bacterium could solve the problem with plasmids (and if it is thinkable then it should have happened)*.

*Then I stumbled across the explanation I put forward in this paper. I personally find this compelling, and in any case it should only be added to the reasons given above. The answer concerns the volume of cytoplasm governed by a single nucleoid, and the number of generations. If a prokaryote were to magically wave into existence multiple copies of the requisite plasmid in exactly the right places, it would still be a prokaryote, with a prokaryotic genome and no intracellular transport networks worth speaking of. It would have all the energy that it needed, but it would be unable to distribute anything about the cell. Until such transport networks evolved, which would presumably take quite a few generations, the bacterium would gain no advantage from being bigger, in which case the plasmid would be unnecessary and lost as superfluous baggage; or the bacterium would expand in size, in which case the plasmid would be useful, but in the absence of intracellular transport networks, which take many generations to evolve, the cell would die anyway. So plasmids do not solve the problem*.

*What about endosymbionts? The critical point here is that endosymbionts govern a bacterial volume of cytoplasm, because they are bacteria. They do not face a transport problem. Slowly, over many generations, as the endosymbionts lose unnecessary genes, the host cell makes energy savings that can in principle be dedicated to the evolution of a dynamic intracellular transport network at no net energetic cost. Bacteria can't do this because they don't have the spare energetic capacity to evolve the genes necessary--they simply get outcompeted by more streamlined competitors. So the key is generations: endosymbiosis is a relatively stable state over hundreds or thousands or billions of generations. Gene loss is the standard outcome of endosymbiont evolution. As the intracellular transport networks evolve, more genes can be lost from the endosymbiont, until finally, over many generations, the endosymbiont genome has become a plasmid-like mitochondrial genome. At all times the host cell was able to distribute resources to all parts of itself, and at all times the energetic price of evolving a dynamic intracellular transport system was paid for in the hard currency of ATP, made available by the slow loss of unnecessary endosymbiont genes over many generations. Thus the resemblance of mtDNA to a plasmid is a mirage. Plasmids cannot solve the supply problem, and full genomes (which have to be copied and inherited using the cell's own segregation and division machinery) are fixed in size and cannot solve the energy problem*.

In summary. Asking 'Why?' questions in evolutionary biology is dangerous, trying to answer them with sweeping hypotheses is dangerous doubly (I certainly realize that I am as guilty as anyone else on this account). Still, this type of risk-taking has to be applauded if we wish to "understand" evolution in any meaningful way. More specifically, I believe that the energetic efficiency argument is important to explain why there are (as far as we know) no primary amitochondrial eukaryotes: symbiosis triggered eukaryogenesis, and allowing a dramatic increase in cell size through efficient energetic was part and parcel of this chain of events. As nicely put by Lane, it was "permissive not prescriptive", i.e., a critical condition but by no account an overarching cause of eukaryogenesis; other factors such as, for example, a massive intron attack on the host genome could have been extremely important as well. I am much more skeptical about the role of energetic consideration as the explanation for the increase of the genome size and gene number in eukaryotes. The extreme genome inflation is a late affair occurring only in some lineages. Granted, this inflation would not have been possible outside the context of the eukaryotic cell but this does not seem to be relevant when eukaryogenesis is considered. The moderate genome expansion seen in unicellular eukaryotes does not require such an explanation, and other factors including the change in regulatory strategies could be much more important.

### Author's response

*I have addressed most of these points already. I have at no point argued that mitochondria drove eukaryogenesis, but I maintain that they were necessary to permit genome sizes above the bacterial maximum of around 10,000 genes and 10 Mb. A question that I did not raise in the paper, but which seems worth mentioning here, is how much genetic experimentation is necessary to evolve complex traits such as phagocytosis? Modern phagotrophs dedicate at least several hundred genes, possibly as many as a couple of thousand, to phagocytosis. Admittedly many of these will be refinements and unnecessary for some rudimentary form of phagocytosis. On the other hand, genes and proteins do not spring fully formed from the head of Zeus. How many failed experiments, proteins expressed but not fully functional, does it take to evolve a completely new trait like phagocytosis? I imagine, but don't know how to get at this question experimentally (or theoretically) that it must take many more than the total number of genes that are required in the end. Ten times as many? I don't know but that doesn't sound unreasonable. I suspect that it requires energy to burn, that cells must not be penalized for profligacy and experimentation with new gene families, new protein folds, new traits, and so on. This is utterly different from simply picking up fully functional operons, which is what prokayotes do. What mitochondria did was give eukaryotes the energetic freedom to experiment with new genes, new traits, new forms of regulation, in a profligate manner, all of which lie beyond the energetic bounds of prokaryotes. This energetic freedom enabled the unparalleled genomic experimentation at the origin of eukaryotes, when according to Koonin's own work, some 2500-3000 new gene families evolved--an extraordinary period of invention which was permitted, but not prescribed, by mitochondria*.

*As an aside, and relevant to Mark van der Giezen's comments below as well, the potentially profligate energy costs of de novo protein evolution for phagocytosis also explains why some phagocytes can do without mitochondria, and with bacterial genome sizes, such as Entamoeba with its 10,000 genes. Yes, it is a phagocyte without mitochondria (they became mitosomes), but this does not mean that phagocytosis can evolve in the absence of energy-transducing mitochondria. Quite the contrary. Entamoeba effectively inherited a cassette of fully functional genes for phagocytosis from a mitochondriate ancestor. All the profligate experimentation with new genes had already been done and dusted. Over many generations it adapted to its new niche, and along the way eliminated unnecessary traits, as parasites usually do. It lost its mitochondria, as they evolved reductively into mitosomes. Critically, as I note in the paper, it also lost major aspects of intermediary metabolism that it could also do without in the context of its parasitic lifestyle. It ended up without mitochondrial power, and lo and behold it fits in beautifully, with 10,000 genes, at around the bacterial maximum genome size in the absence of mitochondria. This is hardly proof, but as circumstantial evidence for the power of mitochondria, it is wonderful*.

*Finally, Koonin notes that other mechanisms, such as massive intron attack, might have been very important, and I wholly concur. I suggest that the combination of a high mutation rate (caused by intron attack) with the elimination of genome size constraints and the loss of the cell wall, enabled cell fusions and genome duplications--the origin of a sexual cell cycle--placing many theoretical ideas on the origin of sex in a specific setting. Again, these ideas can be tested via mathematical models, and work on this has begun*.

### Reviewer 2

William Martin, Heinrich-Heine-Universität, Düsseldorf

This is an exceptional paper. While those who think in categories of branches as the key to understanding eukaryote origin are in a traffic jam of stagnation, those focusing on the origin of mitochondria as the key to the issue find themselves in the fast-lane with good visibility, dry asphalt, and no cars in sight. This paper spearheads progress in understanding what the prokaryote-eukaryote divide is all about, makes progress with tempo, and in exquisite style to boot. I offer a few thought that the author might wish to consider.

### Author's response

Thank you for a wonderful and constructive review!

#### The origin of the eukaryotic cell was a unique event

Para 3: Prokaryotic extinctions. The SOS responses of prokaryotes, like the more facile incorporation of foreign DNA in *E. coli *(MutS down-regulation) or gene transfer agents in proteobacteria exemplify the difference between dinosaurs and prokaryotes in terms of reacting to potential individual-or species-terminating threats. In other words, animals do not have the option of getting foreign genes or mating with new species as a means to avoid extinction. Prokaryotes do.

### Author's response

*Fair point. They also have much smaller population sizes*.

Para 4: the assumed existence of a diverse ....the group was seen

Para 4: however later revealed that all ..... evolution to specialized organelles

#### Eukaryotes originated in an endosymbiosis between prokaryotes

Para 2: ... [47] whatever we would have called an alphaproteobacterium 1.5-2 billion years ago...

Para 3: maybe... albeit different studies suggesting affinities with modern archaebacterial groups. [why cite [46] twice?]

#### Cell fusions best explain the accumulation of eukaryotic traits

Para 3: ...occupied the Archezoan niche. What does that mean? What is an Archezoan niche as opposed to a eukaryotic niche?

#### Energy per gene

Para 7: *Entamoeba *has about 9938 genes (typo 1000)

### Author's response

*I have amended all these in the text*.

#### The issue of scaling

Para 2: Somewhere in this paragraph you could use terms like "concentrations of DNA, ribosomes, proteins" etc. to make it clear what volume is doing. Explain that you adjust the volume first, then worry about chromosome and ribosome copy numbers to compensate. That is the point in scaling, no?

### Author's response

*Yes. Amended*.

Para 3: why is cell division a problem? Explain, not clear to me.

Para 6: ...given that cell division

It seems prudent to consider how giant bacteria actually do divide and some of the incisive thoughts of HP Erickson [79] on this aspect. There are bioenergetic constraints here, too, as Z rings never get bigger than about 1 micron.

### Author's response

*A large bacterium cannot divide using a Z ring, as noted; and a vacuole makes binary fission difficult. Giant bacteria have evolved strikingly different mechanisms of cell division. In the case of Epulopiscium, daughter cells (endospores) grow inside the mother cell; in the case of Thiomargarita, the cell divides reductively to form four daughter cells in much the same way as animal embryos, for which they may have been mistaken in the fossil record. Either way, becoming larger precludes standard binary fission, and so forces the evolution of other mechanisms of cell division*.

*I have added a couple of explanatory sentences to the text*.

#### Only endosymbiosis can fashion giant nuclear genomes

Para 5: This is a beautiful, a spectacular paragraph, maybe split it into two or more, and maybe prime the reader a bit better for what is coming. Maybe make it a section with subheader. The 5% genes for metabolites is one entry to the topic. Maybe a good one, maybe. What you are into is the energetic consequences of specialization of the endosymbiont. If we just transfer genes, but need the proteins in the organelle (requires invention of TIM & TOM) then there is no real savings, because the cost of making the proteins is the same whether made in cytosol or organelle. The genes transferred to the host can also be expressed (at a cost) in the host cytosol, regardless of where the proteins end up. So the real energetic benefit comes from specialization of the organelle towards energetic ends (not reduction in general). Energetic specialization requires the AAC. Reduction is not so much the issue as specialization. Some other numbers come to mind, for *E. coli*.

http://redpoll.pharmacy.ualberta.ca/CCDB/cgi-bin/STAT_NEW.cgi

10 Number ATP to make 1 cell 55 billion ATP

11 Number Glucose molecules consumed 1.4 billion molecules

12 Cell division rate 1 division/30 minutes

They stack up well against your 50 trillion ATPs, or 2 trillion per hour or 1 trillion per 30 minute *E. coli *division. How many endosymbionts are you assuming here??? (ahh, 1000 proteins copies, OK). These are very interesting calculations possibly worth further fleshing out. This is a uniquely original approach to the problem, one that textbooks could use to illustrate what mitochondria have done for us lately.

### Author's response

*I have expanded on this as suggested, in the preceding paragraph. In particular, I agree that the critical factor is specialization. I have emphasized this in the text. As an aside, it would be interesting to compare the energetics of eukaryotic endosymbioses generally with mitochondria in particular. Endosymbionts lose genes, and to a point specialize to live in their intracellular surroundings, but they must always be a burden to their host cell. In energetic terms they are pure cost, as they do not export ATP to their host cell. Loss of function is presumably beneficial to both the host cell and the endosymbiont, and this explains why reductive evolution is common; but if all functional losses are simply transferred to the host cell (eg nucleotide synthesis, amino acid biosynthesis, etc) then there can be no net gain by gene loss because overall protein synthesis (for which read ribosome number) is unchanged*.

*In the case of mitochondria, the export of ATP to the host cell means that they are never pure cost, but the size of the energetic gain depends on the loss of function. This is an interesting distinction between an endosymbiont and an organelle. An endosymbiont, being an independent self-replicating entity, can't lose the traits required for independence, either directly or indirectly (meaning they are provided by the host cell). In contrast, an organelle can be stripped down to almost nothing. This is specialization, as pointed out by Martin and also in the review by Koonin, and appears to be critical*.

Para 5: It is about specialization. Specialization. But specialization via selection. That meshes well with CoRR, too.

#### Genome outposts are required for major expansion of oxidative phosphorylation

Para 1: People always focus on the retention of genomes, and it is just as much about the retention of ribosomes (for the same reasons as CoRR states), and that might be worth mentioning. And do we see plasmids with rrn (ribosomal RNA) operons????? Interesting.

Para 2: Regardless... I would delete that clause, it sounds as if you doubt the strengths of CoRR, which I do not think you do.

### Author's response

*To the three points above: agreed, and text modified accordingly*.

Paras 4-5: Excellent and exquisitely important text.

#### Endosymbiotic gene transfer, mutagenesis and the origin of sex

Para 2: Endosymbiotic gene flow is also backed by comparative genomics, for example plants-cyanobacteria.

Para 4: Yes, that is exactly right. Excellent points.

Paras 4-6: This is interesting.

Para 6: First principles. Hmm. I'm not so sure that we are really back to first principles in this paper. We clearly have a set of very explicit premises about the nature of the host and the symbiont, some processes and some logical consequences from which complexity is permitted (but not prescribed, to lift your prescient abstract text). But when Harold Morowitz goes to first principles, he is in quantum mechanics, the Pauli exclusion principle, the periodic table and so forth. So maybe something a bit more modest here would fit, too.

#### Conclusions

Para 2: "are predicted from first principles"

I disagree. The paper contains logical inferences, not a set of predictions. The inferences unfold in an effortless manner to be sure, and they are all tightly interlocked via the same kind of hard-nosed reasoning (energetic, biochemical, made of molecules, and mechanistic). But first principles? If first principles stays, then please explain in a list what the first principles are, and I think what that will uncover is more accurately described as a set of premises (something assumed or proved as a basis for argument or inference).

Maybe "unfold in effortless inference".

### Author's response

*I have removed the offending phrase 'from first principles', if only because I would not wish to seem immodest. For the record, I don't mean quantum mechanics, but I am exploring what I believe to be the fundamental principles of bioenergetics: why proton gradients are universal (because unlike scalar chemical reactions, which are stoichiometric, gradients allow substoichiometric energy conservation, and this is strictly necessary for the growth of anaerobic chemolithotrophs); and why the requirement for gradients over membranes, controlled by genes, ultimately constraints the evolution of complex life. These are basic principles of bioenergetics and as such are likely to apply to all life everywhere in the universe. Only partly tongue in cheek: aliens will need mitochondria too*.

It was a pleasure to read this paper.

### Author's response

Thank you!

### Reviewer 3

W. Ford Doolittle, Dalhousie University

This is a complex, challenging and ambitious paper. I will leave the bioenergetics to reviewers more competent in that area, and comment or expand on just a few of the Nick Lane's general evolutionary propositions. There's enough in his paper to take up several issues of *Biology Direct*.

### Author's response

Thank you!

Nick Lane argues against there having been a long period for the stepwise acquisition of the many features that distinguish all and every eukaryote from all and every prokaryote. If that time were long, many lineages which had not yet got all of these features might be expected to have diverged, and some should have left survivors. These would be true "archezoa", *sensu *Cavalier-Smith. Since such survivors are not found we might justifiably assume a very rapid evolution of the many eukaryote-defining features, driven by selection and (Lane postulates) an elevated mutation rate, this from "early bombardment of genes and introns from the endosymbiont to the host cell".

This is not a new argument, but it is very well articulated here, nicely cartooned by Figure 2. I myself used to favor the neglected alternative, what I call the "Fourth Domain Hypothesis". With this we would imagine a fourth lineage, diverging below the root at the bottom of the trees in Figure 2, bearing homologs of many of the genes now found in bacteria and archaea but also developing those many distinguishing eukaryotic features, with one of its sub-lineages serving as the host in the initial mitochondrial symbiosis. The added distance down and up the tree would be enough to account for the generally great distance between eukaryote-defining genes and their prokaryotic homologs, only some few of these being still recognizable.

This would be a sort of the reciprocal solution: we don't have to imagine a time-compressing speeding up of the evolution of novelties, but we do have to propose that all the other lineages of the fourth domain went extinct. It's not clear to me why this alternative should be so widely neglected now. Mitch Sogin and Hy Hartman (though not generally in agreement) both used to like the idea. Lane's argument that the fact that lineages that we once thought to be Archezoa seem to have arisen several times and are doing fine makes it even less parsimonious to imagine the extinction of any true Archeozoa is a good one, though.

### Author's response

*I would not say that the idea of a 'fourth domain', by which I understand a primitive amitochondriate eukaryotic lineage dating back to LUCA, now extinct, has been neglected at all. It has been argued forcefully by the likes of Christian de Duve, David Penny, Anthony Poole, Patrick Forterre, Carl Woese, and most engagingly, Frank Harold. Cavalier-Smith, while arguing for a much later origin of eukaryotes these days, still sees the host cell as a phagocyte, albeit possibly quite a rudimentary one before the acquisition of mitochondria. The idea is less popular now than previously, if only because the excellent testable prediction--that representatives of the fourth domain should still be lurking among the archezoa--turned out to be falsified, at least in most people's view. That of course does not rule out their extinction--in fact it demands it--but I am surprised at how little critical thought appears to have been addressed to the extinction argument. I am pleased that Doolittle thinks the argument I put forward is a good one*.

*What I have tried to do here is consider the alternative possibility, as rigorously as I can. It may all be wrong, but at least it leads to a number of quite surprising and testable predictions. One reason that the archezoa hypothesis was good is that it too raised a number of testable predictions, and the phylogenetic and morphological studies that addressed them moved the field along positively. This avenue appears to have dried up, however, and it is time to address alternative possibilities, if only, ultimately, to exclude them too*.

*"We don't have to imagine the time-compressing speeding up of the evolution of novelties." I think the assumption of a clocklike mutation rate that applies to all cells is surely equally imaginary. The phylogenetic problems with long-branch attraction among supposedly early-branching eukaryotes such as microsporidia, which later turned out to be highly derived fungi, shows that the evolution rates of parasites are often elevated. Episodes of 'quantum evolution' have been propounded by Cavalier-Smith and dismissed on phylogenetic grounds by Bill Martin and others. However, in the case of the early eukaryotes, it is hard to see how an essentially parasitic situation could have led to anything other than a fast rate of evolution. To postulate that the mutation rate should remain clock-like through an early bombardment of endosymbiotic DNA, including mobile type II introns, surely defies the imagination*.

I do not think that the "early bombardment of genes and introns from the endosymbiont to the host cell", the kind of enhanced mutation that Lane wants to claim as a sort of necessary side-effect in his scenario, will necessarily fill the bill. If current numts and nupts are the model for bombardment (and we have no other model), how much do we really expect basal mutation rate to be elevated by endosymbiosis? And is it a given that mutation rates in eukaryotes are actually elevated in a way that would speed up the evolution of evolutionary novelty? In a 1998 summary [143], Jan Drake, the Charlesworths and Jim Crow write ...

"Mutation rates in microbes with DNA-based chromosomes are close to 1/300 per genome per replication ... Mutation rates in higher eukaryotes are roughly 0.1-100 per genome per sexual generation but are currently indistinguishable from 1/300 per cell division per *effective *genome (which excludes the fraction of the genome in which mutations are neutral)."

Maybe there is an argument that domain shuffling and gene rearrangements that this bombardment might have especially favored are in fact just what was needed to evolve the new eukaryotic functions, in which case Lane might want to make it more explicitly.

### Author's response

*Insofar as numts and nupts are the model for bombardment, it is certainly not their evolutionary rate of accumulation that matters, as this only reflects germ-line transfers, at least in 'higher' eukaryotes. The actual rates of bombardment in somatic cells and in the chronological ageing of yeast are substantially higher, as several recent studies show *[117,118]. *We must also bear in mind that the rate of gene transfer would presumably have been slowed down by the barrier of the nucleus, eukaryotic chromosome packing and the many checks and balances of the eukaryotic cell cycle. In the absence of such defences, the impact of an early intron or DNA bombardment from the endosymbionts would surely have been greater. For these reasons it is not appropriate to consider the mutation rates of modern eukaryotes, as this is in no way representative of the evolutionary situation. My point is not that eukaryotes have a high mutation rate today (they do not) but that they almost certainly did have one during early eukaryogenesis, especially transpositions and rearrangements; and that the evolution of the nucleus and the cell cycle restored some sort of equanimity*.

*I would agree that domain shuffling and gene rearrangements would be an important facet of a high mutation rate caused by bombardment, and I have added a short discussion on this; I envisage the situation to be similar to that pertaining to plant mitochondria today, which is to say a large number of aberrant recombinations (which can give rise to cytoplasmic male sterility) without necessarily an elevated nucleotide substitution rate. The closest equivalent today might be the remarkably high density of mobile group II introns and other transposable elements in Wolbachia endosymbionts, as reported by Leclercq et al. *[120]*, albeit here the endosymbiont, not the host, is the vulnerable party. As noted in my response to Koonin, a mathematical model incorporating plausible mutation rates might offer a way forward here, and we are working on this*.

Lane argues that both prokaryotes and eukaryotes "speciate profligately". I think this is a bit bold, when we really have no good definition or understanding of the possible meaning of species in prokaryotes and only a limited definition for some eukaryotes. Jeff Lawrence's clever title "Speciation without species" [144] notwithstanding, I'm not sure we *can *have one without the other. Indeed, recently, I've started to wonder whether a case could be made for the difference between eukaryotes and prokaryotes having to do with the different degrees to which species--defined as units on which *species selection *might operate--might exist among them. Good tight species might allow more complex hierarchical selection, which arguably might be how and why the tempo and mode of prokaryotic and eukaryotic evolution seem so different. (Just saying.) It also might be that it is the multi-"species" community--rather than the individual organism--that is the appropriate unit for comparing prokaryote and eukaryote complexities (again, just saying.)

### Author's response

*By speciation I mean no more than the emergence of distinct genetic clusters, groups identifiable by genotype or phenotype, not the biological species concept as propounded by Mayr and others, but something closer to the species definition of Jim Mallet, and indeed dating back to Darwin himself *[55]. *This applies to both prokaryotes and eukaryotes, and I have added a clarification and a citation to Mallet here *[55]. *I don't want to get embroiled in the fierce arguments over the definition or distinction between species, but rather to draw attention again to the fact that there is a tremendous variety of extant discontinuous diversity among prokaryotes and eukaryotes, in both cases dispersed into distinct genetic clusters, albeit with differing tightness. When I say there is a lack of speciation across the prokaryote-eukaryote transition, I mean that the genetic clusters, or species, that do occupy this morphological niche are all derived from more complex eukaryotic ancestors, and therefore invaded this space from 'above', not 'below'*.

Similarly, there is a lot packed in to Lane's claim that "the requirement for sex also implies that the population was small; large stable populations should speciate, as indeed happened immediately after the crystallization of LECA, with a near immediate radiation of the eukaryotic supergroups." To be sure, small populations, which large eukaryotes might of necessity have, are better for fixing neutral or even deleterious traits, like introns, possibly. But the first eukaryotes were surely not large organisms and it's not clear why they should have had smaller populations than their still-prokaryotic sisters. And unless we want to argue that all those eukaryote-specific complex traits that LECA supposedly quickly acquired--phagocytosis, cytoskeleton, endomembrane system and so forth--arose neutrally, it would be easier to see them arising in a large population where selection could be more effective, and perhaps easier still if there were many "species" with such populations, swapping advancements by lateral gene transfer, for all that process is in Lane's view "inherently asymmetric". Of course some of us might argue that many eukaryote-specific complex traits indeed arose neutrally [145], but that's not I think the kind of scenario Lane has in mind here.

### Author's response

*I would see quite a lot of the rudimentary eukaryotic cell structures, but not necessarily more complex traits like phagocytosis, as arising early in eukaryogenesis through the shuffling and accumulation of existing traits--probably not involving the de novo evolution of completely new traits, so much as an exploration of the morphological potential within existing traits, such as endomembrane systems. I see this as a response to novel and heavy selection pressures--therefore not strictly neutral evolution--but not necessarily involving a large amount of de novo genetic invention. So, for example, the loss of the cell wall and the origin of the nuclear membrane and internal membranes like the endoplasmic reticulum, do not require a large amount of de novo invention so much as gene transfer to the nucleus and disruption of normal gene expression, as suggested by Bill Martin and others. Already this would be a recognizable eukaryote with a nucleus, endomembranes, mitochondria and a rudimentary cell cycle--cell fusion, genome doublings, straight chromosomes, chromosomal segregation on a protein scaffold derived from plasmid segregation machinery, etc. This is essentially a rearrangement of existing structures in new morphological space. The de novo inventions required at this point would be relatively limited--spliceosomes, telomeres (both derived from mobile group II introns), dynamic cytoskeleton (derived from bacterial or archaeal homologues), nuclear pore complexes, and some degree of targeting to the mitochondria and other endomembranes, I imagine with a great deal less sophistication than today). More complex traits like phagocytosis seem to have evolved independently on three separate occasions after LECA, which fits in very well with this scenario*.

*I would see this period as the origin of various eukaryotic gene families and signature proteins, but not necessarily in large and sophisticated groups--that may well have happened later, after LECA, but explaining why so many gene families do trace back to LECA. Presumably these gene families arose from prokaryotic proteins by limited rearrangements combined with a release from purifying selection for genome streamlining. Later on, in larger and more stable populations, these nascent gene families expanded and were selected for specific functions in different eukaryotic supergroups*.

*Regarding lateral gene transfer in these early eukaryotes, yes of course it might have been important. I assume that it became less important as the properties of the chimeric cell trended from prokaryotic to eukaryotic. If this period was indeed the origin of the cell cycle and reciprocal meiotic sex, then the question arises, why? My suggestion is that lateral gene transfer alone was insufficient to prevent the death of cells through the accumulation of mutations, because it does not trade in whole genomes. Bacteria such as the radiation-resistant Deinococcus radiodurans apparently survive heavy mutation rates not by lateral gene transfer but by accumulating multiple copies of genes, and this is all that I am proposing. In the case of early eukaryotes, I am suggesting that these additional genomes would have been gained by cell fusion with no more than the conventional benefit of outbreeding in sex, i.e. masking of mutations by undamaged copies*.

*Overall, then, I am not suggesting neutral evolution so much as selection--meaning merely successful avoidance of death--in small populations for some quite rudimentary morphological reorganizations, mostly drawing on existing genes and proteins, but in new patterns permitted by the acquisition of mitochondria and the elimination of the heavy selection pressure to lose genes and proteins common to prokaryotes. I would see LECA as morphologically eukaryotic, showing the beginnings of most eukaryotic gene families (presumably derived from existing archaeal or bacterial genes, perhaps with some degree of domain shuffling and rearrangement, but not in a sophisticated guise), but probably not including genuinely complex traits such as phagocytosis at this point*.

### Reviewer 4

#### Mark van der Giezen, University of Exeter

In this intriguing manuscript, the author posits that the true reason behind the rise of the eukaryotes, or even their raison d'être, is their increased ability to produce the ATP that relaxes the energetic constraints facing prokaryotes and therefore allows eukaryotes to become more complex. This increased production of ATP originates from their mitochondria, a prokaryote which got severely crippled in the process of endosymbiosis. That ATP production is behind the mitochondrial endosymbiosis might sound somewhat similar to other hypotheses about mitochondrial origins but appearances are deceiving. Lane does not discuss the forces that forged the symbiosis but the consequences of that event. In addition, most models of eukaryogenesis presume that the bacterial symbiont was taken up by a protoeukaryote. This is energetically not possible according to Lane's argument. In a convincing and an in-hindsight obvious claim, Lane calculates that it is energetically simply not possible to be a eukaryote without mitochondria. There is just not enough energy available in a prokaryotic cell to produce the energy required to sustain larger cell sizes or evolve phagocytosing capability at the expense of essential ATP producing capability. Using two well known giant bacteria, *Epulopisium *and *Thiomargarita*, Lane shows, using some carefully argued calculations, that these two bacterial species more or less forgot to divide and ended up with several thousand copies of their genome in a massive cell, solely to sustain their energetic demand. In addition, their largeness is perceived rather than real, because their centre is metabolically inactive and everything happens in a thin layer of cytoplasm near the cell membrane.

#### Author's response

Thank you, a very good synopsis!

It is generally understood that the evolution of eukaryotes must have been a rare event and that it only happened once. Successfully that is. Undoubtedly, there must have been other attempts but the hurdles were so high that we only know of one winner, which is the lineage that includes ourselves. The presence of many universal eukaryotic features such as conserved intron positions, straight chromosomes, telomeres, etc. argues for the establishment of all these features very early on in eukaryotic life. There might have been more attempts to become eukaryotic but there are no traces left of such attempts. The unsatisfying thing is that we know of no intermediates and the microfossils that are several billion years old are not that illuminating either.

Lane's hypothesis is well argued and the manuscript well written. However, due to the complexity of what is on offer, some sections might need to be re-read to assess the fullness of what is on offer; at least, I had to. I have, however, some puzzling thoughts left. *Entamoeba *is put forward as an example of a simple eukaryote with only 1000 genes (this must be a typo as there are roughly 10 x more than that). It is 'allowed' to be a eukaryote on energetic grounds as it apparently has 3 orders of magnitude more energy than prokaroytes. This allows it to maintain a costly phagocytotic machinery. However, *Entamoeba *no longer maintains classic mitochondria that produce a lot of ATP. Actually, its mitosomes most likely do not produce any ATP at all! Obviously, this is a derived state but how is this organism capable of maintaining its costly lifestyle if not supported by the copious amounts of ATP supplied by mitochondria? Similarly, *Encephalitozoon cuniculi *has an even smaller genome (~2000 protein coding genes) and a gene density that might be closer to prokaryotic than eukaryotic genomes. Is that gene density a necessity to lower the energy bill in the lack of classic mitochondria? It is intriguing to realise that mitosomes and hydrogenosomes were at the base of our renewed interest in eukaryotic and mitochondrial evolution and still seem to be playing a pivotal role.

#### Author's response

*I am grateful for this very thoughtful response to my paper*.

*Yes, indeed the 1000 genes in Entamoeba was an unfortunate typo, now corrected. I did not intend to suggest that Entamoeba has three orders of magnitude more energy than bacteria. I have deleted this ambiguity. I imagine they have a similar energy per gene. This is precisely because Entamoeba has mitosomes, rather than mitochondria capable of oxidative phosphorylation. I have made the point elsewhere that the acquisition of mitochondria was permissive rather than prescriptive: they enabled (from an energetic point of view) and potentially drove (from a mutational point of view) the evolution of quite a complex eukaryotic common ancestor, LECA. However, LECA was free to evolve in any direction, either towards greater complexity, as happened in many prostists, algae, fungi, plants and animals, or reductively, towards morphological simplicity, as happened in the archezoa, including Entamoeba. A major point of my paper is that this group arose by reductive evolution from more complex ancestors, and in so doing lost both morphological and genetic complexity, and was accordingly free to lose bioenergetic complexity too. Plainly the same process went even further, to an extreme degree, in Encephalitozoon cuniculi, which is small even by prokaryotic standards. I am nowhere suggesting that these reduced eukaryotes have some kind of energetic advantage over prokaryotes; on the contrary, in purely bioenergetic terms they are likely to be at a relative disadvantage. However, they have compensating advantages, such as being able to phagocytose, not known in any prokaryote*.

The point I am making about Entamoeba is that it was able to maintain the relatively costly lifestyle of phagocytosis at least in part because its parasitic lifestyle enabled it to lose various other energetically costly traits, such as amino-acid and nucleotide biosynthesis, along with the protein synthesis required to maintain these pathways. It ended up with a 'bacterial' number of genes (10,000) and presumably a bacterial energy per gene, but quite a different spectrum of genes and traits, enabling it to compete successfully in a 'bacterial niche.'

*I repeat here a point I made in response to Koonin: evolving a complex trait like phagocytosis de novo is a very different matter to inheriting a fully functional suite of genes from more complex ancestors, and then steadily losing unnecessary traits or superfluous genes in a given environment. I have argued that the de novo evolution of phagocytosis requires abundant energy per gene, enabling genetic 'experimentation', that is unavailable to prokaryotes. This energetic preclusion explains why phagocytosis never arose in prokaryotes, and why it apparently arose independently on perhaps three occasions very early in eukaryotic evolution. Once phagocytosis has evolved, it is perfectly possible to lose mitochondria secondarily*.

*A final point on phagocytosis: prokaryotes compete among themselves, ultimately on the basis of replication speed, albeit under a wide range of conditions, permitting large variations in cell size and metabolic versatility. Regardless of this variation, replication speed depends in large part on ATP availability (to the point that initiation of replication depends on the ATP/ADP ratio), which is in general optimal in quite small cells, with high surface-area-to-volume ratio, hence the typically small size of bacteria. Phagocytosis, once it has evolved, breaks this loop, as success no longer depends on replication speed--a phagocyte can simply eat the opposition, and the faster they replicate the more it has to eat. Thus, once phagocytosis has evolved, the terms of the deal change. Energy per gene becomes relatively unimportant, as large cells simply sequester their dinner and digest it later, even using comparatively inefficient processes such as fermentation. Thus the key point of this paper is that prokaryotes are constrained in genome size and morphological complexity by their energetics, to the point that they cannot evolve complex traits such as phagocytosis de novo. Mitochondria eliminated these energetic constraints, allowing the free accumulation of potentially massive amounts of DNA in a central nucleus, the genetic raw material needed for the evolution of larger size and morphological complexity. Having eliminated this barrier, complex traits such as phagocytosis could evolve--could evolve, I emphasize that the energetics are permissive, not prescriptive--which ultimately meant that lifestyle became much more important than energy-per-gene as the major determinant of genome size in eukaryotes*.
